# Unveiling the Hidden Diversity of Rock-Inhabiting Fungi: Chaetothyriales from China

**DOI:** 10.3390/jof6040187

**Published:** 2020-09-24

**Authors:** Wei Sun, Lei Su, Shun Yang, Jingzu Sun, Bingjie Liu, Rong Fu, Bing Wu, Xingzhong Liu, Lei Cai, Liyun Guo, Meichun Xiang

**Affiliations:** 1 State Key Laboratory of Mycology, Institute of Microbiology, Chinese Academy of Sciences, No 3 Park 1, Beichen West Road, Chaoyang District, Beijing 100101, China; sunwei@sibs.ac.cn (W.S.); sulei131@126.com (L.S.); 18111838667@163.com (S.Y.); sunjz@im.ac.cn (J.S.); liubj3@foxmail.com (B.L.); furong16@mails.ucas.ac.cn (R.F.); wubing@im.ac.cn (B.W.); cail@im.ac.cn (L.C.); 2 University of Chinese Academy of Sciences, No.19(A) Yuquan Road, Shijingshan District, Beijing 100049, China; 3 Department of Microbiology, College of Life Science, Nankai University, No.94 Weijin Road, Nankai District, Tianjin 300071, China; liuxz@nankai.edu.cn; 4 Department of Plant Pathology, China Agricultural University, No.2 Yuanmingyuan West Road, Haidian District, Beijing 100193, China; ppguo@cau.edu.cn

**Keywords:** *Anthracina*, *Bradymyces*, *Cladophialophora*, *Exophiala*, extremotolerant, *Knufia*, *Lithohypha*, fungi, *Trichomerium*

## Abstract

Rock-inhabiting fungi (RIF) are nonlichenized fungi that naturally colonize rock surfaces and subsurfaces. The extremely slow growth rate and lack of distinguishing morphological characteristics of RIF resulted in a poor understanding on their biodiversity. In this study, we surveyed RIF colonizing historical stone monuments and natural rock formations from throughout China. Among over 1000 isolates, after preliminary delimitation using the internal transcribed spacer region (ITS) sequences, representative isolates belonging to Trichomeriaceae and Herpotrichiellaceae were selected for a combined analysis of ITS and the nuclear ribosomal large subunit (nucLSU) to determine the generic placements. Eight clades representing seven known genera and one new genus herein named as *Anthracina* were placed in Trichomeriaceae. While, for Herpotrichiellaceae, two clades corresponded to two genera: *Cladophialophora* and *Exophiala*. Fine-scale phylogenetic analyses using combined sequences of the partial actin gene (ACT), ITS, mitochondrial small subunit ribosomal DNA (mtSSU), nucLSU, the largest subunit of RNA polymerase II (RPB1), small subunit of nuclear ribosomal RNA gene (SSU), translation elongation factor (TEF), and β-tubulin gene (TUB) revealed that these strains represented 11 and 6 new species, respectively, in Trichomeriaceae and Herpotrichiellaceae. The 17 new species were described, illustrated for their morphologies and compared with similar taxa. Our study demonstrated that the diversity of RIF is surprisingly high and still poorly understood. In addition, a rapid strategy for classifying RIF was proposed to determine the generic and familial placements through preliminary ITS and nucLSU analyses, followed by combined analyses of five loci selected from ACT, ITS, mtSSU, nucLSU, RPB1, and/or the second subunit of RNA polymerase II gene (RPB2), SSU, TEF, and TUB regions to classify RIF to the species level.

## 1. Introduction

Natural and manmade rock surfaces harbor a high diversity of lichenized and nonlichenized fungi, algae, and bacteria [1,2,3]. This niche harbors a polyphyletic assemblage of stress-tolerant rock-inhabiting fungi (RIF) with phenotypes that converge on a suite of morphological and physiological adaptations. The term rock-inhabiting fungi (RIF) used in recent phylogenetic analyses encompasses their heterogeneity and varied functional and ecophysiological attributes [4,5,6]. These fungi were first recognized as components of the microbiota of desert rock and lithotrophic Antarctic rock niches [7]. Later, studies of staining monuments and buildings by melanized fungal cells revealed a multitude of new species and genera of RIF [8,9,10,11]. In general, RIF share a set of convergent features, including slow-growing, black to olivaceous brown meristematic colonies in nature, thick-walled melanized cells, meristematic growth, often with yeast-like cell proliferation, the formation of multicellular propagules from hyphal fragments, and cells submerged in the substratum during axenic culture. The sexual morphs of RIF are generally unknown, and the reproduction and dispersal depend on the vegetative fragmentation or poorly differentiated conidia-like cells. RIF are considered as oligotrophs and are thought to survive from scarce air or waterborne nutrients that arrive to rock surfaces. These fungi can tolerate wide temperature extremes, irradiation, osmotic stress, and desiccation [12,13,14,15].

Despite being worldwide distributed in natural rock formations [16] and manmade materials [8,11,17,18,19,20], RIF have been overlooked due to their slow growth and inconspicuous morphology [21]. The development of phylogenetic classification by multi-locus analyses has provided powerful tools for revealing the immense phylogenetic breadth and diversity of RIF [11,22,23,24]. Over the past two decades, RIF have attracted the attention of mycologists and material engineers, which led to the discovery of at least 21 new genera and 62 new species (data from MycoBank 2020). Phylogenetic analyses revealed that most RIF belong to two main lineages of Ascomycota, i.e., Dothideomycetes and Eurotiomycetes [3,25], whereas fewer taxa have been reported from the Eurotiomycetes, especially in families Trichomeriaceae and Herpotrihiellaceae in order Chaetothyriales [3,25,26].

The Trichomeriaceae was recently proposed based on phylogenetic analysis of *Trichomerium* [27,28]. *Trichomerium* was described by Spegazzini [29] based on the type species *T. coffeicola* (Puttemans) Speg. occurring on *Roystonea regia* in Cuba. Subsequently, *Trichomerium* was affiliated with the Capnodiaceae [30], then transferred into Triposporiopsidaceae [31] and, eventually, into the Chaetothyriaceae [32] based on sessile ascomata and trans-septate hyaline ascospores. Chomnunti et al. [27,28] proposed a new family of Trichomeriaceae based on the phylogenetic reconstructions of *Trichomerium* and placed the Trichomeriaceae in Chaetothyriales. To date, the genera *Arthrocladium, Bradymyces*, *Knufia*, *Lithohypha, Metulocladosporiella*, and *Trichomerium* have been accepted in the Trichomeriaceae [11,24,33,34]. Although, initially, species in the Trichomeriaceae were recognized as epiphytic sooty molds on living plants and insect honey dew excretions, the epiphytic and rock surface habitats of these fungi were recognized to be overlapped. Since then, some slow-growing, melanized, and poorly differentiated fungal strains from rocks [24] and monuments [11] have been added to the family.

*Exophiala* J.W. Carmich and *Cladophialophora* Borelli are two asexual genera in Herpotrichiellaceae [25,35], of which species were frequently isolated from diverse substrates, such as animal, human, plant, soil, and water, as saprobes or pathogens [33,35,36,37]. Several species of *Exophiala* and *Cladophialophora* were reported as rock-inhabiting fungi [11,24]. *Exophiala* was introduced by Carmichael [38], who used *E. salmonis* as the type of species and was characterized by having an annellidic conidiogenesis producing slimy heads of conidia. *Cladophialophora* Borelli, typified by *C. ajelloi* Borelli, was introduced to accommodate one-celled conidia species that are produced through large phialidic collarettes in differentiated conidial apparatus [39] and long, coherent chains of dry conidia that originate by blastic conidiogenesis [40]. However, the limited morphological characteristics are insufficient to distinguish these species from their close relatives [37,41,42]. Although the diagnostic schemes, including morphological, physiological, serological parameters, and phylogenic approaches, have been applied for those genera, especially for some clinical important species [43,44,45,46], phylogenic analyses provided an efficient and powerful approach to identify this group of fungi [25,33,41,42].

In this study, over 1000 strains of RIF were isolated during an extensive rocks survey in China. After a preliminary examination and classification by ITS (the internal transcribed spacer region) barcoding, representative isolates belonging to Trichomeriaceae and Herpotrichiellaceae were selected for an in-depth analysis. Based on the multi-locus phylogenetic analysis and morphological features, a new genus *Anthracina* and 17 new species were recognized within Chaetothyriales. Thus, within the context of documenting the diversity of RIF in China, this report significantly expands the worldwide diversity of Chaetothyriales and provides descriptions of new taxa.

## 2. Materials and Methods

### 2.1. Sampling Sites and Sampling

**Figure 1:**
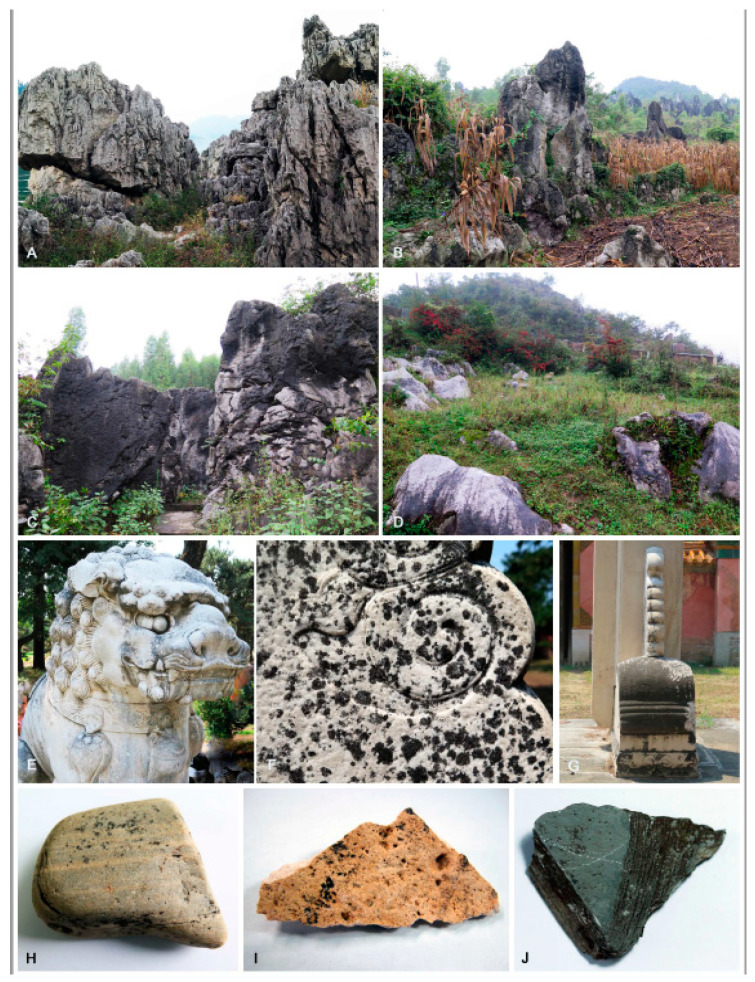
Rock-inhabiting fungi (RIF) colonizing natural rock and historical stone monuments. (**A**–**D**) Representative sampling sites in the Karst landform area in Guizhou Province, (**E**–**G**) RIF colonizing on historical monuments in Beijing, (**H**–**I**) rock with RIF sampled from Tibet, and (**J**) rock with RIF sampled from Guizhou Province.

Samples were collected from a variety of ecosystems in China (Figure 1). These ecosystems are Qinghai-Tibet plateaus, deserts, karst landform, mountains, and historical stone monuments that cross ten provinces: Gansu, Anhui, Yunnan, Hebei, Guizhou, Guangxi, Jiangxi, Henan, Beijing, and Tibet. Rock samples bearing black colonies were taken directly from natural habitats or rock samples that were arbitrarily collected by breaking off rock pieces (>100 g) from rock formations. Samples were sealed in plastic bags and transported to the laboratory at ambient temperatures. For monuments or ornamental stones sample, black yeast-like fungi were isolated from the samples by picking off single fungal colonies with sterile needles and transferring them onto dichloran rose bengal agar (Solarbio, DRBC) in Petri plates (with 100-mg/L streptomycin added after autoclaving) [12].

### 2.2. Isolation of Fungi

Fungi were isolated using previous methods [47], except that surface-disinfected samples were dried on filter papers before processing. Rock samples with black colonies were cut into pieces (2–3 mm^3^) with an industrial stone splitter (Model CM-10, Hydrasplit, Park Industries, Inc., St. Cloud, MN, USA). Rock pieces were surface-disinfected with 95% (*v*/*v*) ethanol for 3–5 s, transferred to physiological saline containing 0.001% (*v*/*v*) tween-20, washed with sterilized distilled water, and dried on sterilized filter papers. About 1 g of each sample was pulverized in a sterilized mortar, and the powder was suspended in 2–3 mL of sterile water. Aliquot of suspensions (200 μL) were spread evenly onto DRBC in Petri plates (with 100-mg/L streptomycin added after autoclaving). Plates were incubated at 10 °C for 4 weeks. Darkly pigmented colonies developing on plates were transferred to malt extract agar (MEA; malt extract 1%, peptone 1‰, glucose 1%, and agar 1.5%) in Petri plates for purification and identification. Type specimens were deposited in the Herbarium of Mycology, Institute of Microbiology, Chinese Academy of Sciences, Beijing, China (HMAS), and ex-type living cultures were deposited in the China General Microbiological Culture Collection Center (CGMCC).

### 2.3. Morphology

Colony morphologies on 2% malt extract agar (MEA; BD, Sparks, MD USA) plates were observed and described after six weeks of incubation at 25 °C. Microscopic morphology was examined using slide cultures [48]. Digital images of samples were acquired using a Nikon Eclipse 80i (Tokyo, Japan) microscope and were digitally optimized with the software NIS-Elements Documentation (v. 4.00.00). The modified slide culture method was applied to observe the sporulation [49]. Pieces of 2% MEA (1 cm × 1 cm) were placed above two layers of sterilized filter paper on the bottom of a Petri dish and kept moisture with sterilized water. The edge of each agar block was inoculated with a small amount of mycelium, and a sterile cover slip was placed over the inoculated agar. After 30–60 days, the cover slide was removed from the agar block and then was placed on a slide with water mounting for morphological observation. At least 20 measurements per structure were conducted under a microscope with differential interference contrast (DIC).

### 2.4. DNA Extraction, PCR Amplification, and Sequencing

Genomic DNA was extracted from fungal mycelia on MEA plates, as previous described [50] and amended with cell disruption by beating for 45 s and 50 s before being incubated at 85 °C in a water bath for 45–60 min. ACT (the partial actin gene), ITS, mtSSU (mitochondrial small subunit ribosomal DNA), nucLSU (the nuclear ribosomal large subunit), RPB1 (the largest subunit of RNA polymerase II), SSU (the small subunit of nuclear ribosomal RNA gene), TEF (translation elongation factor), and TUB (the partial β-tubulin gene) fragments were PCR-amplified with corresponding primers and annealing temperatures (Table 1). PCR amplifications were made from 30-μL reaction mixtures containing 1.0-μL DNA template, 1.0-μL each forward and reverse primers, 15 μL of 2× MasterMix (TIANGEN Co. Ltd. Beijing, China), and 12 μL of H_2_O. The PCR parameters were 94 °C for 40 s, followed by 40 cycles at the temperatures appropriate for the gene amplified (Table 1) for 60 s, 72 °C for 90 s, and a final extension at 72 °C for 10 min. The PCR products were sequenced by SinoGenoMax Co. Ltd. (Beijing, China). The sequenced amplicons were compared with those in the GenBank database via BLAST searching to find the most similar taxonomic designations (Table S1). All novel sequences obtained in this study were deposited in the NCBI GenBank database (Table S1), and the final matrices were used for phylogenetic analyses in TreeBASE (www.treebase.org; accession number: S22289).

### 2.5. Alignment and Phylogenetic Analyses

Sequences of closely related fungi were obtained from GenBank (Table S1) and uploaded to MAFFT version 7 (http://www.ebi.ac.uk/Tools/msa/mafft/) for alignment [60]. Sequence alignments were manually adjusted with MEGA v. 5 [61]. Phylogenetic reconstructions were estimated using maximum likelihood (ML) and Bayesian inference (BI). ML analyses were run with RAXML-HPV v. 0.3 [62]. The RAxML software accommodated the GTR + GAMMA model. For the Bayesian analyses, the models of evolution were estimated by MrModeltest v. 2.3 [63]. Posterior probabilities (PP) [64] were determined by Markov Chain Monte Carlo sampling (BMCMC) in MrBayes 3.0b4 [65] under the estimated model of evolution. Four simultaneous Markov chains were run for 1,000,000 generations, and trees were sampled every 100 generations (resulting in 10,000 total trees). The first 2500 trees, which represented the burn-in phase of the analysis, were discarded, and the remaining 7500 trees were used for calculating PP values in a majority rule consensus tree.

**Figure 2:**
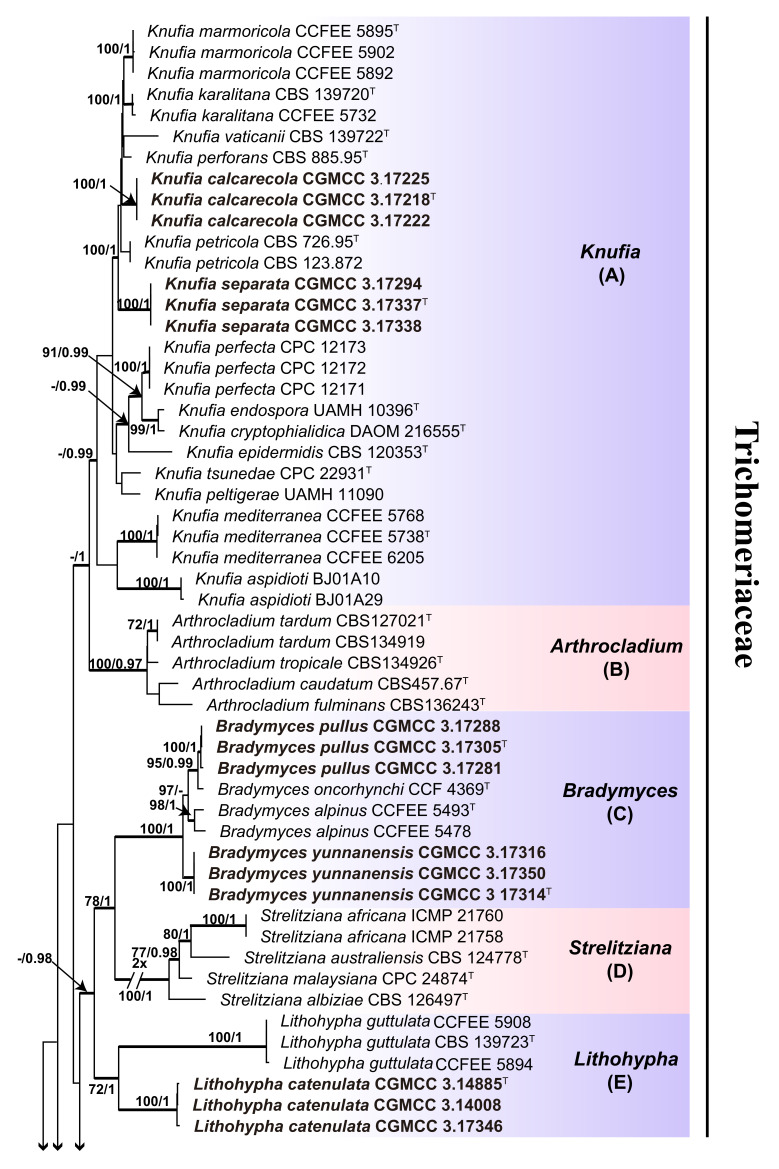

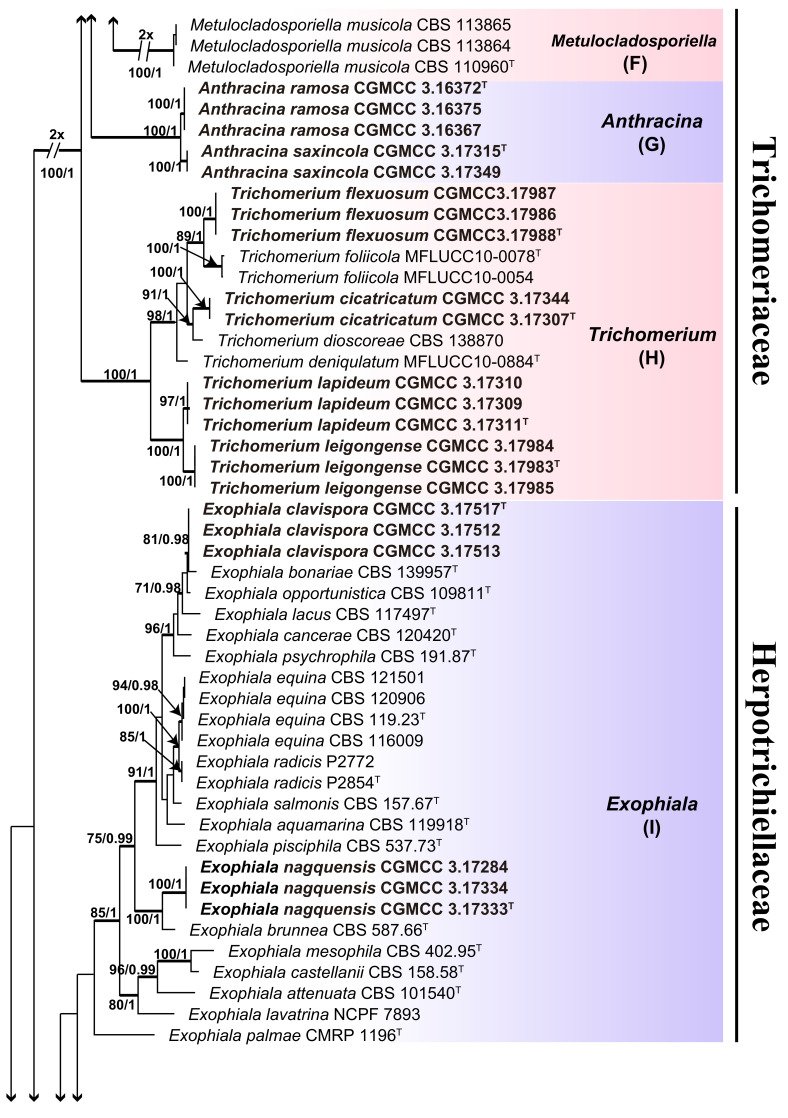

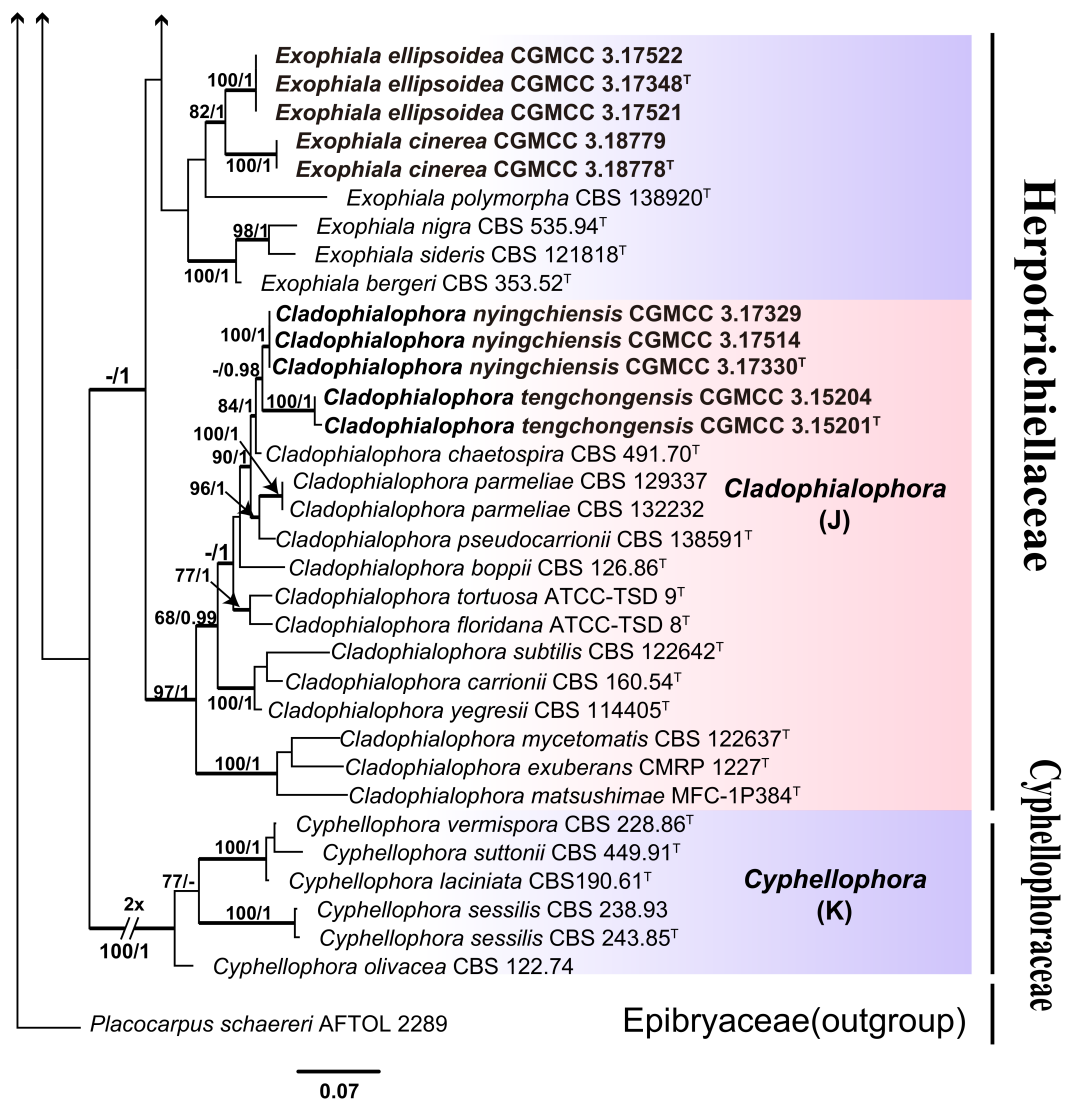
Phylogenetic tree generated by the maximum likelihood analysis using combined sequences of the internal transcribed spacer region (ITS) and partial nuclear ribosomal large subunit (nucLSU) loci of the families Trichomeriaceae and Herpotrichiellaceae. Bootstrap values ≥70% (**left**) and Bayesian posterior probability values ≥0.95 (**right**) are indicated at nodes (maximum likelihood/Bayesian inference or ML/BI). Thickened branches represent posterior probabilities (>0.95) from BI. *Placocarpus schaereri* strain AFTOL 2289 is used as the outgroup. Novel sequences generated in this study are indicated in bold. Ex-type cultures are marked with ”**^T^**”.

**Figure 3:**
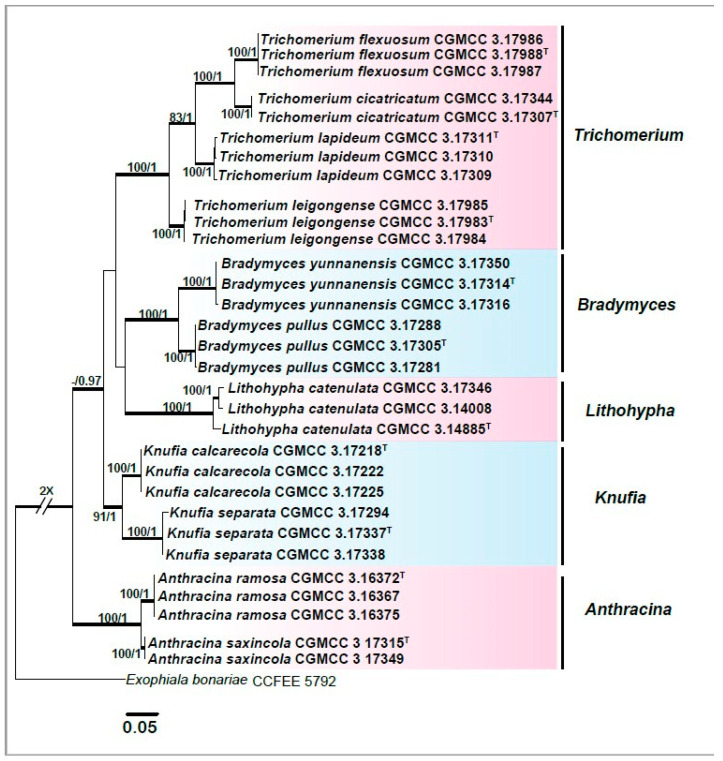
Phylogenetic tree generated by the maximum likelihood analysis using combined sequences of ITS, nucLSU, mitochondrial small subunit ribosomal DNA (mtSSU), the largest subunit of RNA polymerase II (RPB1), and the partial β-tubulin gene (TUB) loci of the family Trichomeriaceae. Bootstrap values ≥70% (**left**) and Bayesian posterior probability values ≥0.95 (**right**) are indicated at nodes (ML/BI). Thickened branches represent posterior probabilities (>0.95) from BI. Novel sequences generated in this study are indicated in bold. *Exophiala bonariae* strain CCFEE 5792 is used as the outgroup. Ex-type cultures are marked with “**^T^**”.

To build a phylogenetic framework (Figure 2), the concatenated alignment of ITS (1–862) and nucLSU (863–1771) regions consisted of 1771 characters for 136 strains. All characters were assessed to be unordered and equally weighed. Missing data were treated as question marks. Based on the Akaike Information Criterion (AIC) criteria, evolution models, including a GTR + I + G with ingamma-distributed rates for ITS and nucLSU, were selected for the partitioned Bayesian inference. To resolve species boundaries, the dataset of combined sequences (3745 characters, including gaps) of five loci, including ITS (1–635), nucLSU (636–1484), mtSSU (1485–2180), RPB1 (2181–2845), and TUB (2846–3745), were aligned and used for phylogenetic reconstruction for 32 taxa, including the outgroup taxon, *Exophiala bonariae* CCFEE5792 (Figure 3). Ambiguous regions and introns were delimited manually and excluded from the alignments. RPB1 and TUB genes that failed to amplify from some isolates were replaced by question marks. Based on the AIC criteria, the evolution models, including a GTR + I + G with inverse ingamma-distributed rates for ITS, TUB, and RPB1; a GTR + I + G with propinv-distributed rates for nucLSU; and a GTR + G with gamma-distributed rates for mtSSU, were selected for the partitioned Bayesian inference. Trees were visualized in FigTree ver. 1.4.3 (http://tree.bio.ed.ac.uk/software/Fig. tree/). Maximum likelihood bootstrap proportions (MLBP) ≥ 70% and Bayesian inference posterior probabilities (BIPP) ≥ 0.95 are indicated at nodes.

**Figure 4:**
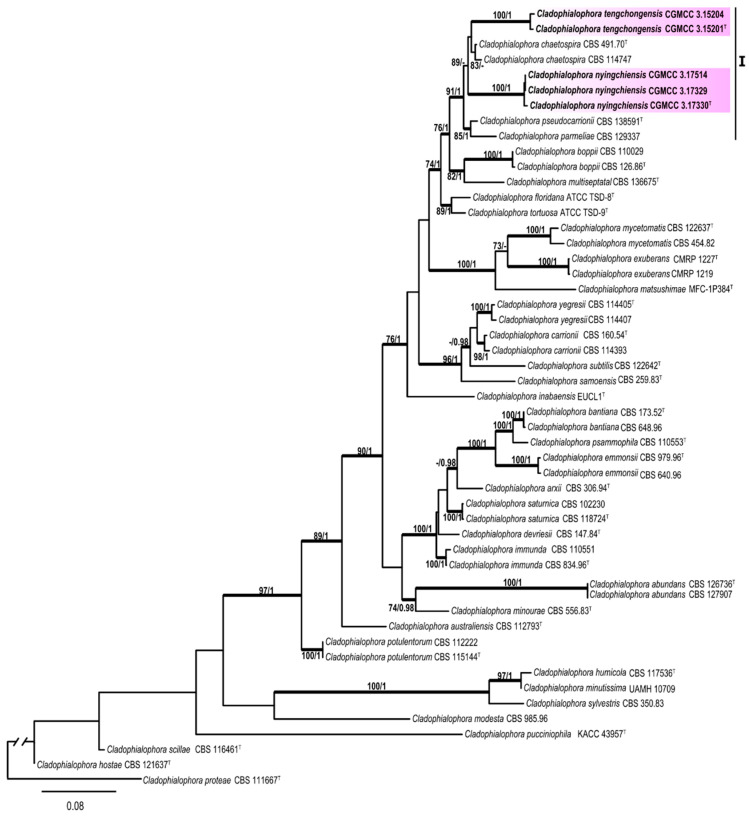
Phylogenic tree generated by the maximum likelihood analysis using combined sequences of ITS, small subunit of nuclear ribosomal RNA gene (SSU), translation elongation factor (TEF), TUB, and nucLSU loci of the genus *Cladophialophora*. Bootstrap values ≥70% (**left**) and Bayesian posterior probability values ≥0.95 **(right**) are indicated at nodes (ML/BI). Thickened branches represent posterior probabilities (>0.95) from BI. Novel sequences generated in this study are indicated in bold. *Cladophialophora proteae* strain CBS 111667 is used as the outgroup. Ex-type cultures are marked with “**^T^**”.

**Figure 5:**
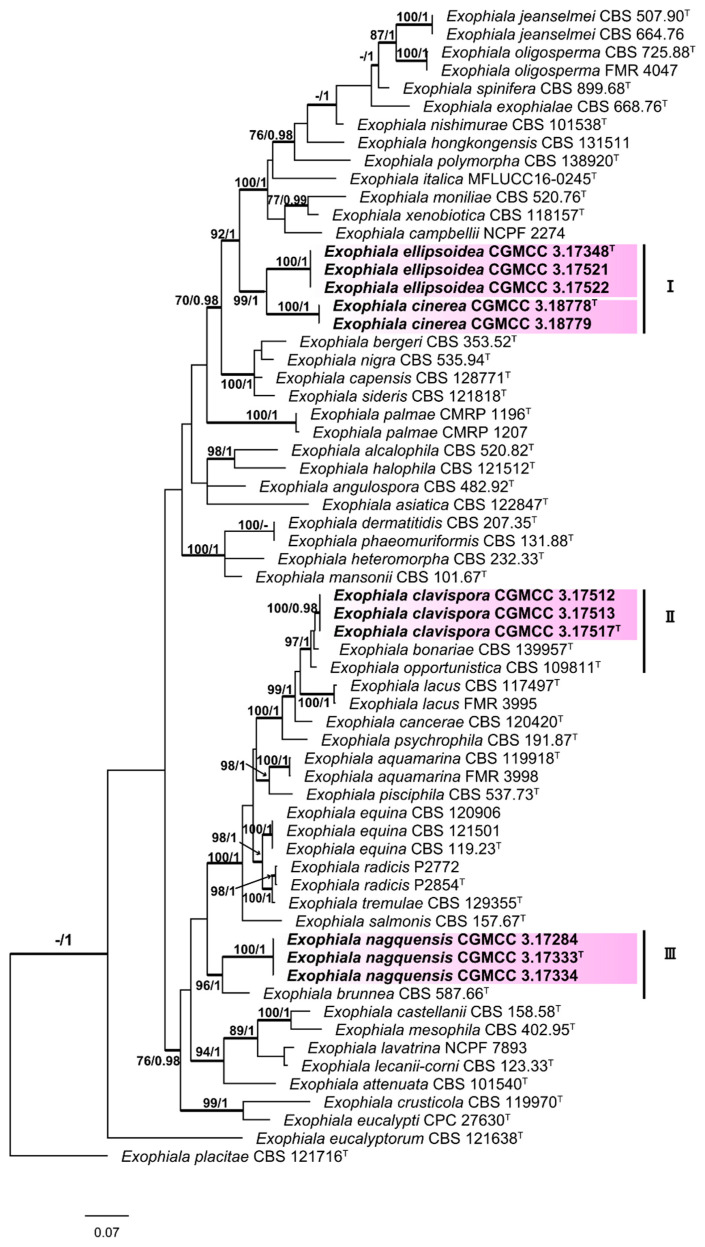
Phylogenic tree generated by the maximum likelihood analysis using combined sequences of ITS, SSU, TEF, TUB, and the partial actin gene (ACT) loci of the genus *Exophiala*. Bootstrap values ≥70 (**left**) and Bayesian posterior probability values ≥0.95 (**right**) are indicated at nodes (ML/BI). Thickened branches represent posterior probabilities (>0.95) from BI. Novel sequences generated in this study are indicated in bold. *Exophiala placitae* strain CBS 121716 is used as the outgroup. Ex-type cultures are marked with “**^T^**”.

For *Cladophialophora*, the 5-locus sequence alignment (ITS: 1–703 characters, SSU: 704–2420 characters, TEF: 2421–2658 characters, TUB: 2659–3167, and nucLSU: 3168–4066 characters) for a total of 51 taxa was analyzed, selecting *C. proteae* as the outgroup. For the Bayesian analyses, HKY + I + G was selected as the best fit model for SSU and TUB, SYM+G for TEF, SYM + I + G for ITS, and GTR + I + G for nucLSU. The phylogenetic tree is shown in Figure 4. For *Exophiala*, the multi-locus sequence alignment (ITS: 1–750 characters, SSU: 751–2456 characters, TEF: 2457–2711 characters, TUB: 2712–3173 characters, and ACT: 3174–3716 characters) of the total 64 taxa was analyzed using *E. placitae* as the outgroup. For the Bayesian analyses, GTR + I + G was selected as the best model for ACT, TEF, and ITS and HKY + I + G for EF and TUB. The phylogenetic tree is shown in Figure 5.

## 3. Results

### 3.1. Multi-Locus Phylogeny

The Bayesian and ML trees resulted in similar topologies—two distinct in Trichomeriaceae and Herpotrichiellaceae, as well as robustly supported monophyletic clades in the order Chaetothyriales—were evident (Figure 2).

The phylogenic tree based on a combined sequences of the ITS and nucLSU indicated that the selected 47 isolates (in bold) were distributed among 11 independent clades, of which six are from *Knufia* (clade A), *Bradymyces* (clade C), *Lithohypha* (clade E), *Trichomerium* (clade H), *Exophiala* (clade I), and *Cladophialophora* (clade J), respectively. We propose that clade G, clearly delineated with a high bootstrap value, represents a new genus in the Trichomeriaceae of the order Chaetothyriales (Figure 2). We propose a new genus, *Anthracina*, for clade G and describe the morphological characteristics of two new species in the genus (Figure 2).

The phylogenetic tree constructed from the five-locus dataset (ITS, nucLSU, mtSSU, RPB1, and TUB) suggested that subclades nested within each primary clade could be resolved into distinct species. After detailed observations of morphological features, those subclades were considered as new species. The proposed new genus, *Anthracina,* was comprised of two new species (MLBP/BIPP = 100%/1.00), designated as *A. ramosa* (MLBP/BIPP = 100%/1.00) and *A. saxincola* (MLBP/BIPP = 100%/1.00). The *Bradymyces* clade contained two new species (MLBP/BIPP = 100%/1.00), designated as *B. pullus* (MLBP/BIPP = 100%/1.00) and *B. yunnanensis* (MLBP/BIPP = 100%/1.00). The *Lithohypha* clade contained one new species, designated as *L. catenulata* (MLBP/BIPP = 100%/1.00). The *Trichomerium* clade formed four subclades corresponding to four new species (MLBP/BIPP = 100%/1.00), designated as *T. cicatricatum* (MLBP/BIPP = 100%/1.00), *T. flexuosum* (MLBP/BIPP = 100%/1.00), *T. lapideum* (MLBP/BIPP = 100%/1.00), and *T. leigongense* (MLBP/BIPP = 100%/1.00). The *Knufia* clade (MLBP/BIPP = 91%/1.00) contained two new species, designated as *K. calcarecola* (MLBP/BIPP = 100%/1.00) and *K. separata* (MLBP/BIPP = 100%/1.00) (Figure 3).

The phylogenetic tree (Figure 4) showed that the sampled specimens clustered into one subclade (I) corresponded to two new species, *Cladophialophora nyingchiensis* and *C. tengchongensis*, with well-supported bootstrap values. Morphological characteristics also supported the establishment of those species. Subclade I contained five species (MLBP/BIPP = 91%/1.00), including the newly described *C. tengchongensis* (MLBP/BIPP = 100%/1.00) and *C. nyingchiensis* (MLBP/BIPP = 100%/1.00).

The results of the phylogenic analysis shown in Figure 5 revealed that sampled specimens clustered into three subclades (I–Ⅲ) corresponded to four new species (*Exophiala ellipsoidea*, *E. cinerea*, *E. clavispora*, and *E. nagquensis*), with well-supported bootstrap values. The newly described *E. ellipsoidea* (MLBP/BIPP = 100%/1.00) clustered with *E. cinerea* (MLBP/BIPP = 100%/1.00); these two species constituted the subclade I (MLBP/BIPP = 99%/1.00). In subclade Ⅱ (MLBP/BIPP = 97%/1.00), five strains clustered into three groups, including newly described *E. clavispora* (MLBP/BIPP = 100%/0.98), *E. bonariae*, and *E. opportunistica*. Subclade Ⅲ contained two species (MLBP/BIPP = 96%/1.00), including newly described *E. nagquensis* (MLBP/BIPP = 100%/1.00) and *E. brunnea*. Morphological characters, as reported in the following Taxonomy section, also support the establishment of these new species.

### 3.2. Taxonomy

Novel genus and species belonging to phylum Ascomycota, subphylum Pezizomycotina, class Eurotiomycetes, order Chaetothyriales, family Trichomeriaceae:

Trichomeriaceae

*Anthracina* L. Su, W. Sun and M.C. Xiang, gen. nov.

MycoBank MB810883

Etymology: anthracina (Lat., fem. adj.), referring to the coal-black color of the colonies.

Colonies on MEA are slow-growing, with minimal expansion on agar media, black to dark brown, velvety, and with irregular margin. Hyphae branched, composed of irregular, hyaline to light-yellow cells. Swollen cells intercalary or terminal. Conidiogenesis, if present, results in endoconidia from vegetative cells. 

Type of species: *Anthracina ramosa* L. Su, W. Sun and M.C. Xiang

Notes: Phylogenetic analyses based on the combined two or five loci strongly supported the establishment of the new genus in Trichomeriaceae (Figure 2 and Figure 3).

*Anthracina ramosa* L. Su, W. Sun and M.C. Xiang, sp. nov.

**Figure 6:**
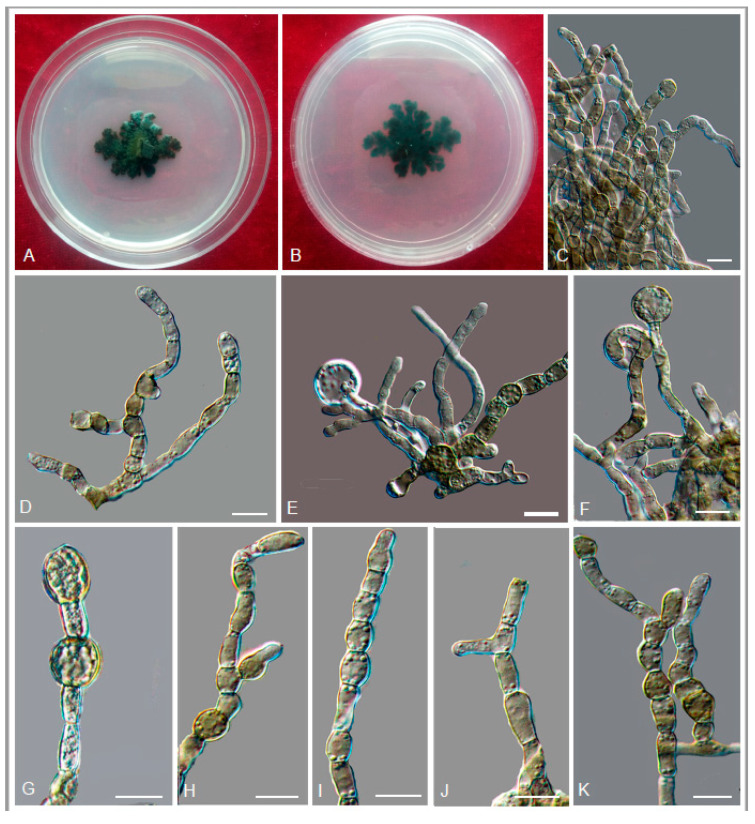
*Anthracina ramosa* (CGMCC3.16372). (**A**,**B**) Colony forward and reverse after 20 weeks on malt extract agar (MEA). (**C**,**D**,**H**–**K**) Moniliform and cylindrical celled hyphae. (**E**–**G**) Terminal and intercalary swollen cells. Scale bars (**C**–**K**) = 10 µm.

MycoBank MB810884 (Figure 6)

Etymology: *ramosa* (Lat., fem. adj.), referring to the frequently branched hyphae.

Type: China: Henan Province: Nanyang City, Xichuan County, 33°09′ N, 111°29′ W, 757 m a.s.l., from limestone in karst formations, 14 October 2012, Lei Su, (HMAS245363 (dried culture)—holotype and CGMCC 3.16372—ex-type culture).

Description: Hyphae moniliform, branched, smooth, 2.2–5.4-μm ($\bar{x}$ = 3.9 μm, *n* = 20)-wide, enteroblastically proliferating, hyaline at first, yellowish brown in age (Figure 6C,D,H–K). Swollen cells spherical to ellipsoidal, 8.6–17.8 μm ($\bar{x}$ = 13.4 μm, *n* = 10) in diam., formed intercalarily or terminally (Figure 6E–G).

Culture characteristics: Colonies on MEA growing slowly, attaining 14-mm-diam. after 20 weeks at 25 °C, greyish-brown, lobate margin irregular, aerial mycelia scant, and velvety; black in reverse (Figure 6A,B). Minimum 4 °C, optimum at 20–25 °C, and maximum 30 °C.

Additional specimens examined: China: Henan Province: Nanyang City, Xichuan County, 33°09′ N, 111°29′ W, 757 m a.s.l., from limestone in karst formations, 14 October 2012, Lei Su, living culture CGMCC 3.16367. China: Gansu Province: Dunhuang City, Dunhuang Desert, 40°09′ N, 94°40′ W, 2435 m a.s.l., from limestone, 6 October 2010, Lei Su, living culture CGMCC 3.16375.

*Anthracina saxincola* L. Su, W. Sun and M.C. Xiang, sp. nov.

**Figure 7:**
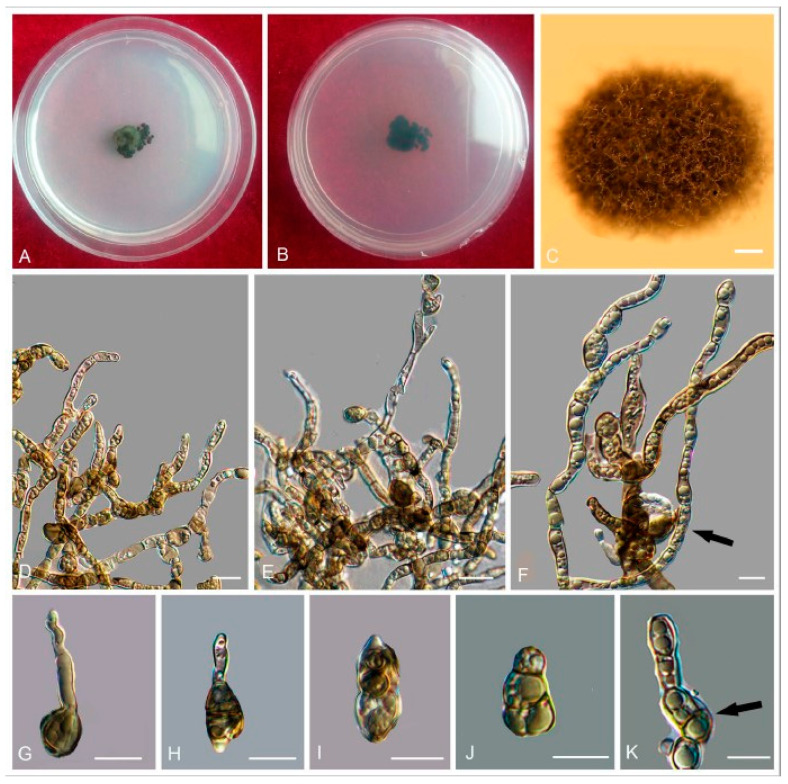
*Anthracina saxincola* (CGMCC3.17315). (**A**–**C**) Colony forward and reverse after 20 weeks on MEA. (**D**,**E**) Different hyphal morphologies. (**F**) Swollen cell (arrow). (**G**) Unicellular body with hypha. (**H**–**J**) Solitary, enlarged, dark-pigmented multicellular bodies. (**K**) Endoconidia in intercalary cells of hyphae (arrow). Scale bars (**C**) = 50 µm and (**D**–**K**) = 10 µm.

MycoBank MB810885 (Figure 7)

Etymology: *saxincola* (Lat., fem. adj.), referring to a stone inhabitant.

Type: China: Yunnan Province: Lijiang City, Yulong Naxi Autonomous County, Jade Dragon Snow Mountain, plateau landscape, 27°16′ N, 100°11′ W, 3250 m a.s.l., from sandstone, 15 January 2013, Lei Su, (HMAS245365 (dried culture)—holotype and CGMCC 3.17315—ex-type culture).

Description: Hyphae moniliform, branched, pale brown to dark brown, 0.8–5.5-μm-wide ($\bar{x}$ = 2.7 μm, *n* = 20), abundant oil drops in young hyphae, enteroblastically proliferating, pale brown at first, yellowish brown when aged (Figure 7D,E). Swollen cell (Figure 7F, arrow) spherical to ellipsoidal, 9.8–16.4 μm in diam. ($\bar{x}$ = 14.5 μm, *n* = 10), formed by intercalary or terminal. Unicellular (Figure 7G) and multicellular bodies (Figure 7H–J) globose to ellipsoidal, brown, 5.2–9.2 × 9.2–15.8 μm ($\bar{x}$ = 6.8 × 13.5 μm, *n* = 10). Endoconidia (arrow in Figure 7K) were occasionally observed in enlarged cells or in multicellular bodies.

Culture characteristics: Colonies on MEA extremely slow-growing, attaining 4-mm-diam. after 20 weeks at 25 °C, compact, center-raised, deep olive-gray to olive-black, with scant, velvety aerial mycelia, flat and glossy near the periphery; black in reverse (Figure 7A–C). Minimum 4 °C, optimum at 20–25 °C, and maximum 30 °C.

Additional specimens examined: China: Yunnan Province: Lijiang City, Yulong Naxi Autonomous County, Jade Dragon Snow Mountain, plateau landscape, 27°16′ N, 100°11′ E, 3250 m a.s.l., from sandstone, 15 January 2013, Lei Su, living culture CGMCC 3.17349.

Notes: The cylindrical, ampulliform, and obovate hyphae of *A. saxincola* apparently differ from other genera, such as *Knufia*, *Bradymyces*, and *Trichomerium* [22,24,27,28,32], in the same family. *A. ramosa* and *A. saxincola* were clustered into a clade with strong bootstrap support (MLBP/BIPP = 100%/1.00) (Figure 2). However, the growth rate of *A. ramosa* is much faster (14 mm in 20 weeks on MEA at 25 °C) than that of *A. saxincola* (4 mm in 20 weeks on MEA at 25 °C). *A. saxincola* also produced multicellular bodies in the aged cultures, and endoconidia were also occasionally observed in enlarged cells or in multicellular bodies, while those were not observed in the culture of *A. ramosa*.

*Bradymyces pullus* L. Su, W. Sun and M.C. Xiang, sp. nov.

**Figure 8:**
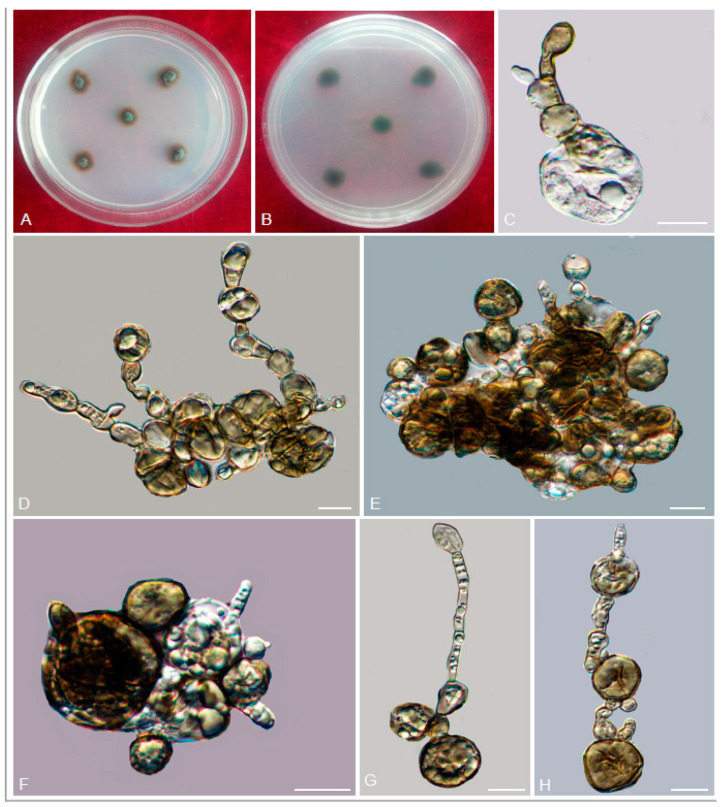
*Bradymyces pullus* (CGMCC 3.17305). (**A**,**B**) Colony forward and reverse after 20 weeks on MEA. (**C**,**E**,**F**) Solitary, enlarged, darkly pigmented multicellular bodies. (**D**) Moniliform hyphae with a mass of differentiated blastic proliferating hyphae. (**G,H**) Terminal and intercalary swollen cells. Scale bars: (**C**–**H**) = 10 µm.

MycoBank MB810888 (Figure 8)

Etymology: *pullus* (Lat., masc. adj.), referring to the black appearance of colonies.

Type: China: Tibet: Shigatse City, Tingri County, Qinghai-Tibet plateau, 28°02′ N, 87°58′ E, 4950 m a.s.l., from granite, 1 July 2012, Xingzhong Liu, (HMAS 245353 (dried culture)—holotype and CGMCC 3.17305—ex-type culture).

Description: Hyphae 2.1–4.9-μm-wide ($\bar{x}$ = 3.4 μm, *n* = 20), brown to medium brown, guttulate, cylindrical, and moniliform parts frequently produced by enteroblastic proliferation (Figure 8D). Swollen cells globose to sub-globose, 7.9–21.6-μm-diam. ($\bar{x}$ = 13.1 μm, *n* = 10) (Figure 8G,H). Multicellular bodies globose, ellipsoidal or irregularly shaped, brown to dark brown, 8.6–16.3-μm ($\bar{x}$ = 14.2 μm, *n* = 10)-wide, developing intercalarily and terminally on the hyphae (Figure 8C,E,F). Endoconidia not observed.

Culture characteristics: Colonies on MEA growing slowly, attaining 6-mm-diam. after 20 weeks at 25 °C, brown to black, with velvety, scant, aerial mycelia, glossy, and irregular margin; black in reverse, commonly with dark-brown crusts and excoriations on the surface (Figure 8A,B). Minimum 4 °C, optimum at 20–25 °C, and maximum 28 °C.

Additional specimens examined: China: Tibet: Nyingchi County, Golug Town, Qinghai-Tibet Plateau, 30°46′ N, 91°36′ E, 4641 m a.s.l., from conglomerate, 3 August 2013, Meichun Xiang, living culture CGMCC 3.17288 and ibid., living culture CGMCC 3.17281.

Notes: This new species is phylogenetically close to *B. oncorhynchi* (MLBP/BIPP = 95%/0.99) (Figure 2). However, the growth rate of *B. pullus* is much slower (6 mm in 20 weeks on MEA at 25 °C) than that of *B. oncorhynchi* (5–8 mm in 42 day on MEA at 25 °C), and *B. oncorhynchi* also produces endoconidia, which were not observed in *B. pullus* [24].

*Bradymyces yunnanensis* L. Su, W. Sun and M.C. Xiang, sp. nov.

**Figure 9:**
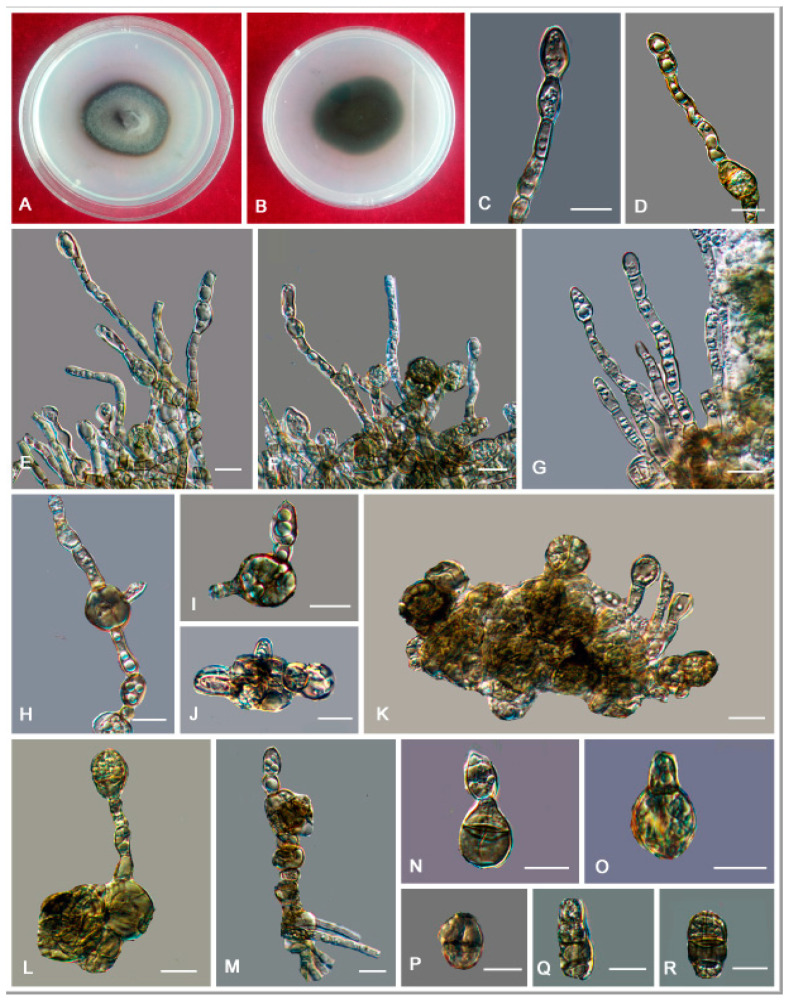
*Bradymyces yunnanensis* (CGMCC 3.17314). (**A**,**B**) Colony forward and reverse after 20 weeks on MEA. (**C**–**G**) Catenated, moniliform hyphae. (**H**,**I**) Swollen cells in the intercalary of hyphae. (**J**–**R**) Multicellular bodies formed from hyphae. Scale bars: (**C**–**R**) = 10 µm.

MycoBank MB810889 (Figure 9)

Etymology: *yunnanensis* (Lat., masc. adj.), referring to the type locality.

Type: China: Yunnan Province: Lijiang City, Yulong Naxi Autonomous County, Jade Dragon Snow Mountain, 27°16′ N, 100°11′ E, 3250 m a.s.l., from sandstone, 15 January 2013, Lei Su, (HMAS 245371 (dried culture)—holotype and CGMCC 3.17314—ex-type culture).

Description: Hyphae smooth, septate with constrictions at the septa, cylindrical yellowish brown to medium brown, 2.5–5.8-μm ($\bar{x}$ = 3.3 μm, *n* = 20)-wide (Figure 9C–G). Swollen cells globose to sub-globose, 6.9–16.5-μm-diam. ($\bar{x}$ = 12.1 μm, *n* = 10) (Figure 9H,I). Multicellular bodies globose, ellipsoidal, or irregularly shaped, medium brown to dark brown, 9.9–16.3 × 14.5–22.6 μm ($\bar{x}$ = 12.9 × 16.5 μm, *n* = 10), formed terminally or intercalarily in old cultures (Figure 9J–R). Minimum 4 °C, optimum at 20–25 °C, and maximum 30 °C.

Additional specimens examined: China: Yunnan Province: Lijiang City, Yulong Naxi Autonomous County, Jade Dragon Snow Mountain, 27°16′ N, 100°11′ E, 3250 m a.s.l., from sandstone, 15 January 2013, Lei Su, living culture CGMCC 3.17316 and ibid., living culture CGMCC 3.17350. 

Notes: *B. yunnanensis* can be distinguished from the phylogenetically closed species *B. alpinus* (95% identity in ITS and 99% in nucLSU) by that *B. alpinus* is unable to grow above 25 °C and is usually endoconidia present in one swollen cell. Multicellular bodies of *B. yunnanensis* were more ellipsoidal (9.9–16.3 × 14.5–22.6 μm), while that of *B. alpinus* were more global (10–15-μm-diam.) [24].

*Knufia calcarecola* L. Su, W. Sun and M.C. Xiang, sp. nov.

**Figure 10:**
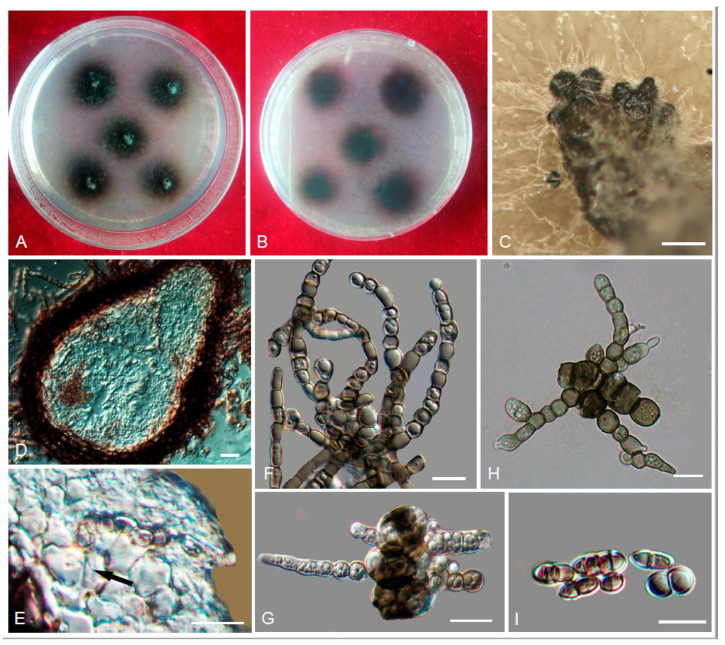
*Knufia calcarecola* (CGMCC 3.17218). (**A**,**B**) Colony forward and reverse after 20 weeks on MEA. (**C**) Pycnidia with hyaline surface hyphae. (**D**) Vertical section through pycnidium. (**E**) Conidiogenous cells (arrow). (**F**) Catenated, moniliform hyphae. (**G**) Multicellular bodies. (**H**) Conidia germination. (**I**) Conidia. Scale bars: (**C**) = 50 μm, (**D**) = 5 μm, and (**E**–**I**) = 10 μm.

MycoBank MB810892 (Figure 10)

Etymology: *calcarecola* (Lat., neut. adj.) referring to limestone from which the type was isolated.

Type: China: Beijing City: Beijing Botanical Garden, dry climate with fog and haze weather in autumn and winter, 39°59′ N, 116°12′ E, 89 m a.s.l., ornamental rocks, from limestone, 12 April 2011, Lei Su, (HMAS 245385 (dried culture)–holotype and CGMCC 3.17218–ex-type culture).

Description: Hyphae catenulate, becoming moniliform with age, septate, constricted at the septa, 3.5–7.1-μm ($\bar{x}$ = 5.3 μm, *n* = 20)-wide, thick-walled, pale brown to greyish brown, guttulate, enteroblastically proliferating (Figure 10F). Pycnidia initials arising from a few irregular cells formed by repeated division of a few hyphae cells, 90.2–186.8-μm-wide, 127.2–232.9-μm-high ($\bar{x}$ = 140.2 × 200.6 μm, *n* = 10), sub-globose to globose, brown to dark–brown (Figure 10C,D). Conidiogenous cells hyaline to pale brown, cylindrical to sub-globose, 6.9–8.2 × 12.3–23.2 μm ($\bar{x}$ = 8.4 × 16.5 μm, *n* = 10) (Figure 10E). Conidia gemmating proliferation is commonly present laterally along the hyphae (Figure 10H). Conidia hyaline, usually aseptate, occasionally 1-septate, constricted at the septa, smooth-walled, cylindrical to clavate, 4.8–6.2 × 8.5–14.1 μm ($\bar{x}$ = 5.3 × 10.8 μm, *n* = 20) (Figure 10I). Terminal multicellular bodies ellipsoidal to muriform, brown to dark brown, 7.3–21.4 × 10.4–22.6 μm ($\bar{x}$ = 18.5 × 19.4 μm, *n* = 10) (Figure 10G).

Culture characteristics: Colonies on MEA growing slowly, attaining 15-mm-diam. after 20 weeks at 25 °C, compact, raised centrally, greyish brown, aerial mycelia sparse to scant, black in reverse (Figure 10A,B). Minimum 4 °C, optimum at 20–25 °C, and maximum 28 °C.

Distribution: Beijing (China).

Additional specimens examined: China, Beijing, the west campus of China Agricultural University, ornamental rocks, 40°01′ N, 116°16′ E, 56 m a.s.l., from limestone, 1 November 2010, Lei Su, living cultures CGMCC 3.17225; ibid., CGMCC 3.17222.

Notes: K. calcarecola is phylogenetically close to *K. petricola*. However, pycnidia were only observed in K. calcarecola.

*Knufia separata* L. Su, W. Sun and M.C. Xiang, sp. nov.

**Figure 11:**
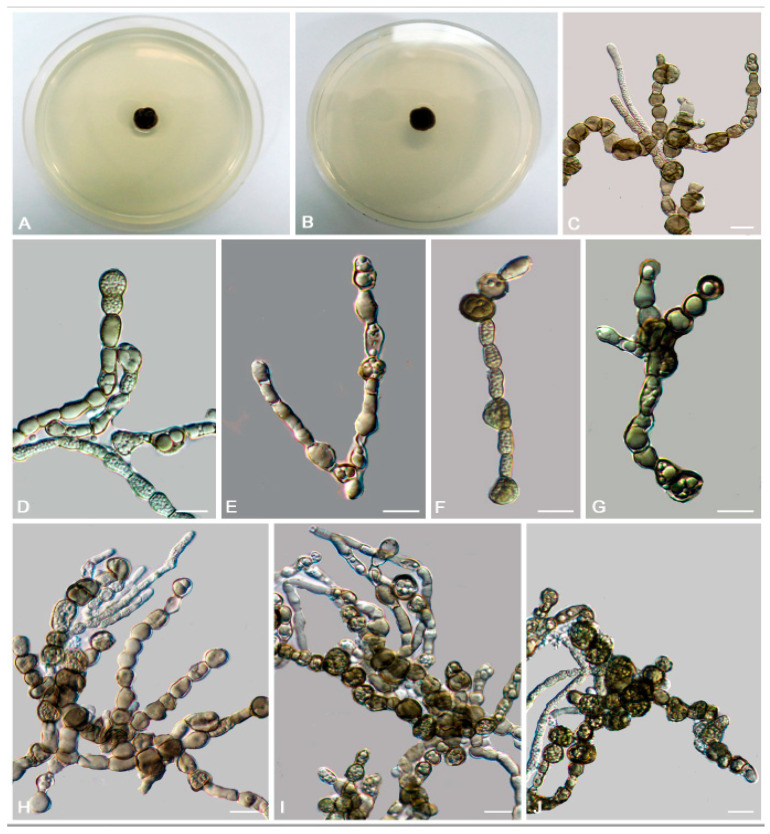
*Knufia separata* (CGMCC 3.17337). (**A**,**B**) Colony forward and reverse after 4 weeks on MEA. (**C**–**E**) Differentiated hyphae. (**F**,**G**) Swollen cell. (**H**–**J**) Dark pigmented multicellular bodies. Scale bars: (**C**–**J**) = 10 µm.

MycoBank MB810894 (Figure 11)

Etymology: *separata* (Lat., neut. adj.) referring to regular separation of hyphae into cylindrical cells with age.

Type: China: Tibet: Naqu County, Qinghai-Tibet Plateau, 31°06′ N, 91°42′ E, 4762 m a.s.l., from gravel in alpine cold and hypoxia, dry climate, 7 August 2013, Meichun Xiang, (HMAS245361 (dried culture)–holotype and CGMCC 3.17337–ex-type culture).

Description: Hyphae moniliform, branched, 2.6–6.9-μm-diam. ($\bar{x}$ = 4.8 μm, *n* = 20), hyaline to pale brown, smooth or verruculose, consisting of broadly ellipsoidal to cylindrical cells, apically and laterally budding cells forming dense and dark aggregates (Figure 11C–E). Swollen cells globose to sub-globose, 6.0–16.8-μm-diam. ($\bar{x}$ = 13.6 μm, *n* = 10) (Figure 11F,G). Multicellular bodies globose, ellipsoidal, or irregular, dark brown, 7.4–12.3 × 6.2–11.2 μm ($\bar{x}$ = 9.7 × 8.4 μm, *n* = 10), intercalary multicellular bodies developing the hyphae (Figure 11H–J).

Culture characteristics: Colonies on MEA growing slowly, attaining 3-mm-diam. after four weeks at 25 °C, blackish-brown to olivaceous black, with a velvety, aerial short hyphae, regular margin, black in reverse (Figure 11A,B). Minimum 4 °C, optimum at 20–25 °C, and maximum 29 °C.

Distribution: Naqu County (Tibet China).

Additional specimens examined: China: Tibet: Naqu County, Qinghai-Tibet Plateau, 31°06′ N, 91°42′ E, 4762 m a.s.l., from gravel in alpine cold and hypoxia, dry climate, 7 August 2013, Meichun Xiang, living culture CGMCC 3.17338 and ibid., CGMCC 3.17294.

Notes: The difference between *K. separata* and *K. karalitana* is the growth rate on MEA, with the latter growing faster (8 mm in three weeks) than the former (3 mm in four weeks) [11].

*Lithohypha catenulata* L. Su, W. Sun and M.C. Xiang, sp. nov.

**Figure 12:**
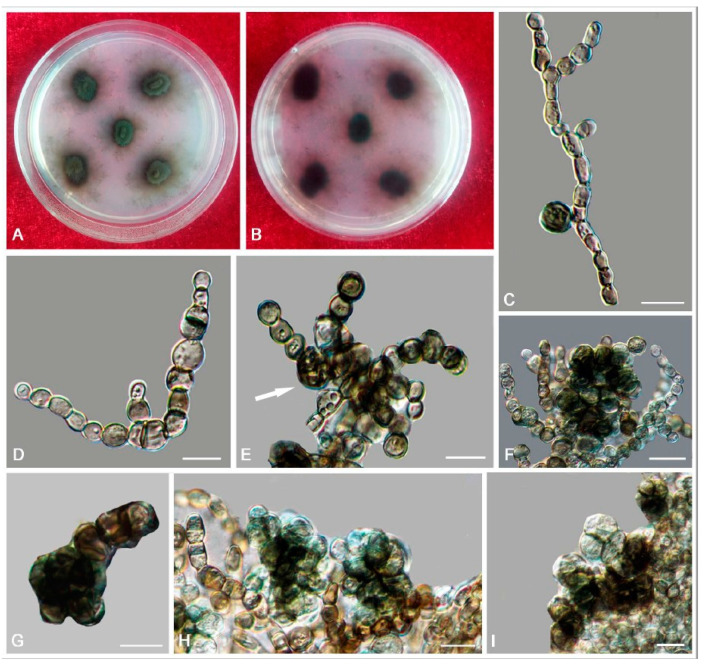
*Lithohypha catenulata* (CGMCC 3.14885). (**A**,**B**) Colony surface and reverse after 20 weeks on MEA. (**C**,**D**) Catenated, moniliform hyphal cells proliferating often at the apex. (**E**) Swollen cells with dark brown fragmented crusts on their surface (arrows). (**F**–**I**) Solitary, enlarged, dark pigmented multicellular bodies with transverse and longitudinal septa. Scale bars: (**C**–**I**) = 10 µm.

MycoBank MB810886 (Figure 12)

Etymology: *catenulata* (Lat., neut. adj.) referring to catenate roundish cells forming moniliform hyphae.

Type: China: Beijing: the west campus of China Agricultural University, 40°00′ N, 116°12′ E, 108 m a.s.l., from ornamental limestone, 12 April 2011, Lei Su, (HMAS264536 (dried culture)–holotype and CGMCC 3.14885–ex-type culture).

Description: Hyphae moniliform, branched, smooth, pale brown to brown, diffusely guttulate, consisting of spherical to subspherical cells with enteroblastic proliferation (Figure 12C,D). Swollen cells globose to sub-globose, frequently intercalary, terminal or lateral, 6.1–10.2 μm ($\bar{x}$ = 7.5 μm, *n* = 20) in young cultures, pale brown, gradually turning dark brown, forming dark brown fragmented crusts on the colony surface (Figure 12E). Multicellular bodies global to ellipsoidal, brown to dark brown, with transverse and longitudinal septa, widening up to 21.4 μm ($\bar{x}$ = 16.8 μm, *n* = 10) (Figure 12F–I).

Culture characteristics: Colonies on MEA growing slowly, attaining 18-mm-diam. after 20 weeks at 25 °C, blackish brown to black, velvety, with sharp and irregularly lobed margin; black in reverse (Figure 12A,B). Minimum 4 °C, optimum at 20–25 °C, and maximum 31 °C.

Additional specimen examined: China: Beijing: the west campus of China Agricultural University, 40°00′ N, 116°12′ E, 108 m a.s.l., from ornamental limestone, 12 April 2011, Lei Su, living culture CGMCC 3.14008; China, Hebei Province, Zhangjiakou City, Yuxian County, Xiaowutai Mountain, 40°23′ N, 114°51′ E, 1011 m a.s.l., from limestone, 24 October 2013, Lei Su, living culture CGMCC 3.17346.

Notes: Morphologically, *L. catenulate* share some similar characters with *L. guttulata* in having multicellular bodies. However, *L. catenulate* has transverse and longitudinal septa multicellular bodies up to 21.4 μm wide.

*Trichomerium cicatricatum* L. Su, W. Sun and M.C. Xiang, sp. nov.

**Figure 13:**
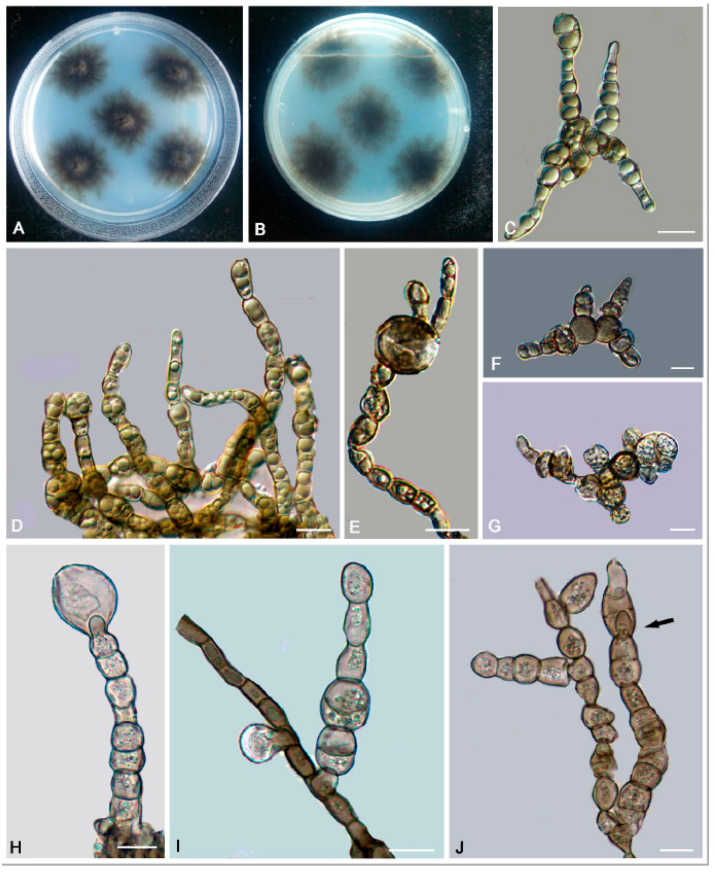
*Trichomerium cicatricatum* (CGMCC 3.17307). (**A**,**B**) Colony forward and reverse after 20 weeks on MEA. (**C**) Conidia with 4 arms. (**D**–**F**) Moniliform hyphae with proliferation at the apex. (**G**) Solitary, enlarged, darkly pigmented multicellular body. (**H**–**J**) Endoconidia develops within intercalary and terminal cells (arrow). Scale bars: (**C**–**J**) = 10 µm.

MycoBank MB810890 (Figure 13)

Etymology: *cicatricatum* (Lat., masc. adj.), referring to scars at conidial base after secession.

Type: China: Jiangxi Province: Jiujiang City, Guling Town, Lushan Mountain, from sandstone, climate with abundant rainfall and sunshine, 29°36′ N, 115°59′ E, 455 m a.s.l., 19 May 2013, Lei Su, (HMAS245352 (dried culture)–holotype and CGMCC 3.17307–ex-type culture).

Description: Hyphae cylindrical, branched, septate, constricted at the septa, 2–6-μm-wide, brown to dark brown, oil drops abundant in young hyphae, enteroblastic proliferation common at the ends of moniliform hyphae, terminal cells frequently larger than the subterminal cells, 14.1–16.9-μm-long, 4.9–7.0-μm-wide ($\bar{x}$ = 15.2 × 6.5 μm, *n* = 5), often proliferating with umbonate apex due to budding (Figure 13D–F). Endoconidia rarely present, unicellular, globose, sub-globose to ellipsoidal, 6.3–15.2 ($\bar{x}$ = 12.5 μm, *n* = 5)-μm-diam., light brown, usually 1-endoconidia present in individual swollen intercalary or terminal cells (Figure 13H–J, arrow). Multicellular bodies brown to dark brown, 6.9–14.9 × 10.2–16.5 μm ($\bar{x}$ = 10.5 × 13.6 μm, *n* = 10) (Figure 13G). Conidia *Tripospermum*-like, pale brown to dark brown, consisting of a subcylindrical basal cell, 1–3-septate, 20.9–33.6 × 3.2–7.5 ($\bar{x}$ = 27.6 × 5.6 μm, *n* = 5), with a truncate hilum, giving rise to three to four lateral arms from a central cell, arms one to two septate, subcylindrical with obtusely rounded ends, 18.5–27.8 × 2.2–7.5 ($\bar{x}$ = 22.7 × 4.9 μm, *n* = 5) (Figure 13C).

Culture characters: Colonies on MEA growing slowly, attaining 13-mm-diam. after 20 weeks at 25 °C, compact, slightly raised at the center, pale black, with scarce, short, velvety aerial mycelia; margin irregular; black in reverse (Figure 13A,B). Minimum 4 °C, optimum at 20–25 °C, and maximum 28 °C.

Additional specimen examined: China: Jiangxi Province: Jiujiang City, Guling Town, Lushan Mountain, from sandstone, climate with abundant rainfall and sunshine, 29°36′ N, 115°59′ E, 455 m a.s.l., 19 May 2013, Lei Su, living culture CGMCC 3.17344.

Notes: Phylogenetic analyses and high bootstrap support values indicated that *T. cicatricatum* is closely related to *T. dioscoreae* (MLBP/BIPP = 91%/1.00) (Figure 2). However, *T. dioscoreae* causes leaf spots with faster growing mycelium and smaller conidia (10–20 × 3–5 µm) [66] compared to *T. cicatricatum.*

*Trichomerium flexuosum* W. Sun, X.Z. Liu and M.C. Xiang, sp. nov.

**Figure 14:**
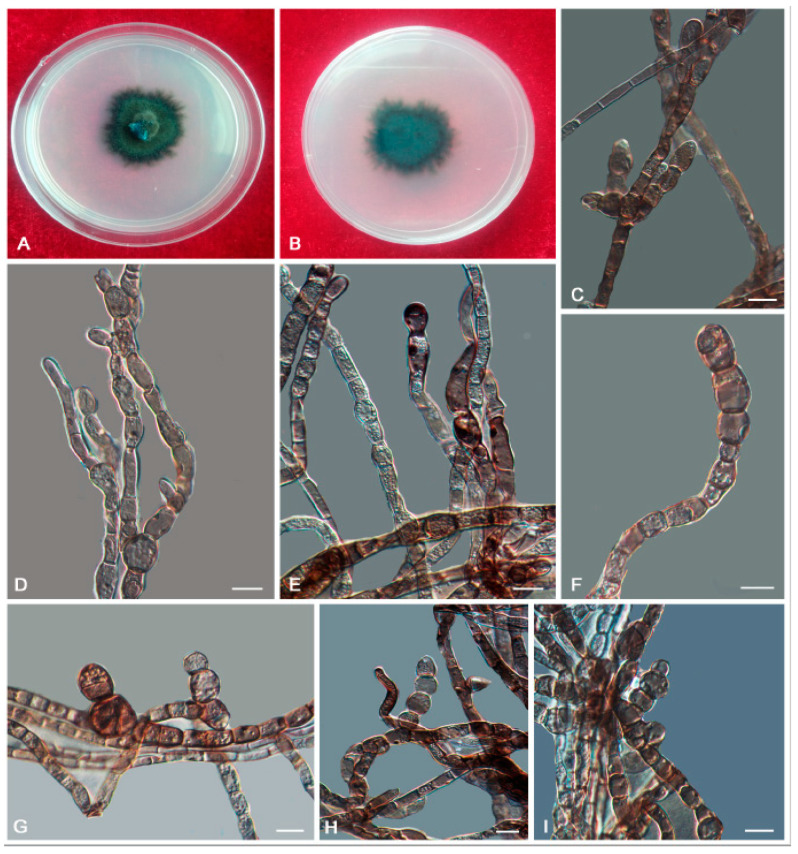
*Trichomerium flexuosum* (CGMCC 3.17988). (**A**,**B**) Colony forward and reverse after 20 weeks on MEA. (**C**,**D**) Hyphae with septa constricted. (**E**,**F**) Apical swollen cell. (**G**–**I**) Moniliform hyphae with blastic proliferation of the branches. Scale bars: (**C**–**I**) = 10 µm.

MycoBank MB 817689 (Figure 14)

Etymology: *flexuosum* (Lat., masc. adj.) referring to the flexuous hyphae.

Type: China: Guizhou Province: Tongren City, Fanjing Mountain, 27°54′ N, 108°41′ E, 2313 m a.s.l., from rock in vegetation-free area, 12 November 2014, Meichun Xiang, (HMAS 246982 (dried culture)–holotype and CGMCC 3.17988–ex-type culture).

Description: Hyphae cylindrical, branched, smooth, constricted at the septa, 2.9–7.8-μm-wide ($\bar{x}$ = 4.7 μm, *n* = 20), dark brown, guttulate, enteroblastically proliferating (Figure 14C,D). Swollen cells globose, ellipsoidal, or irregular, brownish to brown, developing in the hyphal apices or intercalary, up to 10.8-μm-wide ($\bar{x}$ = 8.7 μm, *n* = 10), apical blastic proliferation commonly in moniliform hyphae (Figure 14E,F). Terminal cells or intercalary cells, 5.0–8.5-μm-long, 7.3–9.0-μm-wide ($\bar{x}$ = 6.8 × 8.2 μm, *n* = 10), often proliferating with umbonate apices and branched by budding (Figure 14G–I).

Culture characters: Colonies on MEA growing slowly, attaining 15-mm-diam. after 20 weeks at 25 °C, dark brown, velvety, with long, grayish-brown aerial hyphae, black irregular margin, black in reverse (Figure 14A,B). Minimum 4 °C, optimum at 20–25 °C, and maximum 29 °C.

Distribution: Tongren City (Guizhou Province, China).

Additional specimen examined: China: Guizhou Province: Tongren City, Fanjing Mountain, 27°54′ N, 108°41′ E, 2313 m a.s.l., from rock in vegetation-free area, 12 November 2014, Meichun Xiang, living culture CGMCC 3.17986 and ibid., living culture CGMCC 3.17987.

Notes: Phylogenetic analyses and high bootstrap support values indicated that *T. flexuosum* is closely related to *T. foliicola* (MLBP/BIPP = 89%/1.00) (Figure 2). However, *T. foliicola* is known to produce a sexual morph, which remains unknown for *T. flexuosum*. The growth of *T. flexuosum* is slower than that of *T. foliicola* [27].

*Trichomerium lapideum* L. Su, W. Sun and M.C. Xiang, sp. nov.

**Figure 15:**
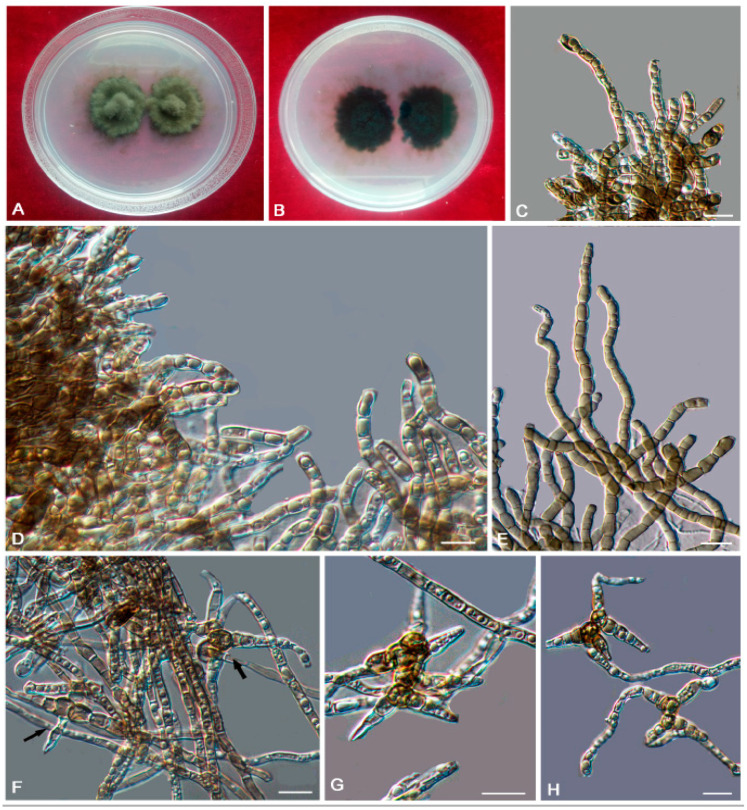
*Trichomerium lapideum* (CGMCC 3.17311). (**A**,**B**) Colony forward and reverse after 20 weeks on MEA. (**C**–**E**) Moniliform hyphae with constricted septa. (**F**–**H**) Branched conidia (arrows). Scale bars: (**C**–**H**) = 10 µm.

MycoBank MB810891 (Figure 15)

Etymology: *lapideum* (Lat., masc. adj.) referring to the type was isolated from hard rock.

Type: China: Jiangxi Province: Jiujiang City, Guling Town, Lushan Mountain, 29°35′ N, 115°59′ E, 952 m a.s.l., from granite in tropical climate, 19 May 2013, Lei Su, (HMAS245387 (dried culture)–holotype and CGMCC 3.17311–ex-type culture).

Description: Hyphae cylindrical, branched, constricted at the septa, dark brown, 2.6–4.9-μm-wide ($\bar{x}$ = 3.8 μm, *n* = 10) (Figure 15C–E). Conidia *Tripospermum*-like, pale brown to dark brown, consisting of a subcylindrical basal cell, 1–3-septate, 12.5–19.8 × 2.2–4.8 ($\bar{x}$ = 15.8 × 3.8 μm, *n* = 5), with truncate hilum, giving rise to three lateral arms, arms one to two septate, subcylindrical with obtusely rounded ends, 7.8–13.5 × 2.2–4.8 ($\bar{x}$ = 10.5 × 3.9 μm, *n* = 5) (Figure 15F–H).

Culture characters: Colonies on MEA slow-growing, attaining 22-mm-diam. after 20 weeks at 25 °C, dark brown to black, slightly raised in the center, with a flat margin, aerial mycelia sparse; black in reverse (Figure 15A,B). Minimum 4 °C, optimum at 20–25 °C, and maximum 29 °C.

Additional specimen examined: China: Jiangxi Province: Jiujiang City, Guling Town, Lushan Mountain, 29°35′ N, 115°59′ E, 952 m a.s.l., from granite in tropical climate, 19 May 2013, Lei Su, living culture CGMCC 3.17309 and ibid., living culture CGMCC 3.17310.

Notes: Phylogenetic analyses showed that *T. lapideum* is the closely related species to *T. deniqulatum*. *T. lapideum* can be distinguished from *T. deniqulatum* in having *Tripospermum*-like conidia, whereas *T. deniqulatum* has ascostromata with sub-globose to globose, brown, 154–175-μm-diam., 163–180-μm-high [27].

*Trichomerium leigongense* W. Sun, L. Su and M.C. Xiang, sp. nov.

**Figure 16:**
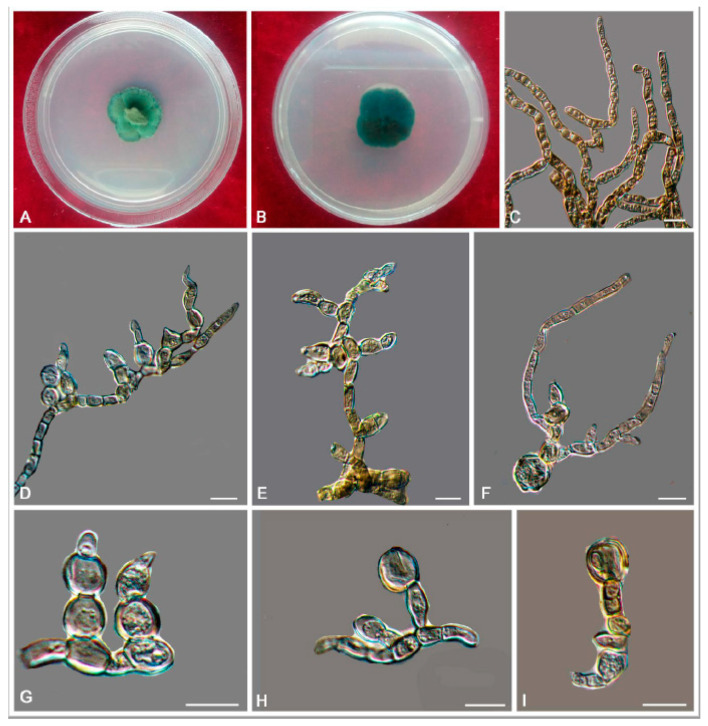
*Trichomerium leigongense* (CGMCC 3.17983). (**A**,**B**) Colony forward and reverse after 20 weeks on MEA. (**C**) Cylindrical, septate hyphae with constricted septa. (**D**–**G**) Moniliform hyphae with blastic proliferation on the lateral branches. (**H**,**I**) Apical unicellular body. Scale bars: (**C**–**I**) = 10 µm.

MycoBank MB 817688 (Figure 16)

Etymology: *leigongense* (Lat., masc. adj.), referring to the type locality.

Type: China: Guizhou Province: Leishan County, Leigong Mountain, 26°23′ N, 108°12′ E, 2112 m a.s.l., from rock surrounded by vegetation, 13 October 2014, Meichun Xiang, (HMAS 246977 (dried culture)–holotype and CGMCC 3.17983–ex-type culture).

Description: Hyphae cylindrical, occasionally toruloid, branched, septate, constricted at the septa, 1.9–4.8 μm ($\bar{x}$ = 3.3 μm, *n* = 20), yellowish brown to mid-brown, the lateral branched moniliform hyphae normally developed by enteroblastic proliferation (Figure 16C–G). Swollen cells globose, ellipsoidal, or sub-globose, light brown to yellowish brown, produced at the hyphal apex, 6.5–7.4-μm-long, 5.3–9.8-μm-wide ($\bar{x}$ = 6.8 × 7.5 μm, *n* = 10) (Figure 16H,I).

Culture characters: Colonies on MEA growing slowly, attaining 21-mm-diam. after 20 weeks at 25 °C, regular margins, dark brown to black, covered by a sparse, velvety, aerial mycelium, black brown in reverse (Figure 16A,B). Minimum 4 °C, optimum at 20–25 °C, and maximum 28 °C.

Distribution: Leishan County (Guizhou Province, China).

Additional specimen examined: China: Guizhou Province: Leishan County, Leigong Mountain, 26°23′ N, 108°12′ E, 2112 m a.s.l., from rock surrounded by vegetation, 13 October 2014, Meichun Xiang, living culture CGMCC 3.17984 and ibid., living culture CGMCC 3.17985.

Notes: *T. leigongense* (CGMCC3.17983) phylogenically resembles *T. lapideum* (CGMCC3.17311) (96% identity in ITS, 99% in nucLSU, 70% in RPB1, and 90% in TUB) (Figure 3). However, swollen cells (6.5–7.4 μm × 5.3–9.8 μm) were present in *T. leigongense* but lack in *T. lapideum*, while Tripospermum-like conidia were present in *T. lapideum* but not in *T. leigongense*. 

Novel species belonging to phylum Ascomycota, subphylum Pezizomycotina, class Eurotiomycetes, order Chaetothyriales, and family Herpotrichiellaceae.

Herpotrihiellaceae

*Cladophialophora nyingchiensis* W. Sun, L. Su, M.C. Xiang and X.Z. Liu, sp. nov.

**Figure 17:**
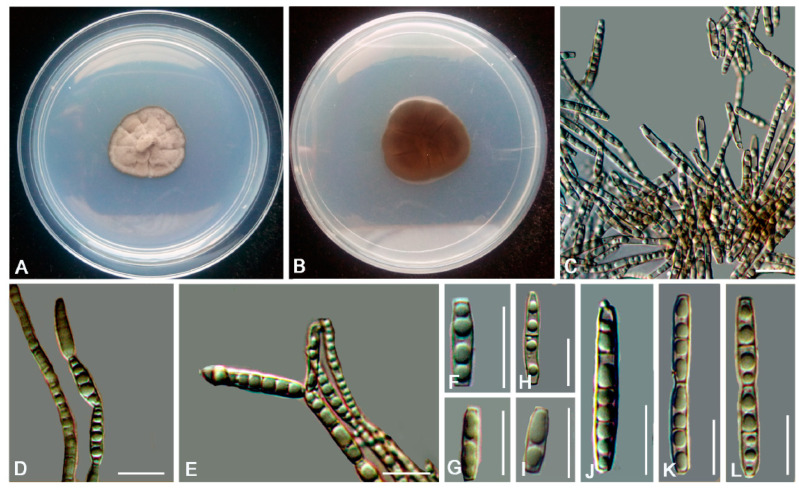
*Cladophialophora nyingchiensis* (CGMCC 3.17330). (**A**,**B**) Forward and reverse of colony on MEA. (**C**–**E**) Conidial chains. (**F**–**L**) Conidia. Scale bars: (**C**–**L**) = 10 µm.

MycoBank MB 824204 (Figure 17)

Etymology: *nyingchiensis* (Lat., masc. adj.), referring to the type locality.

Type: China: Tibet: Nyingchi, Qinghai-Tibet Plateau, 29°45′ N, 94°45′ E, 3448 m a.s.l. from rock, 2 August 2013, Meichun Xiang, (HMAS245367 (dried culture)–holotype and CGMCC 3.17330–ex-type culture).

Description: Mycelium merged into the agar. No diffusible pigment produced. Yeast cells absent. Hyphae smooth and thin-walled, septate, hyaline to pale olivaceous, guttulate, 1.0–2.5-μm-wide. Conidiophores lateral or terminal, pale olivaceous, smooth and thin-walled, ellipsoidal, sub-cylindrical, 8.7–11.2 × 1.3–3.2 μm, erect (Figure 17C–E). Conidia 0-1-septate, formed in long, occasionally branched chains, subhyaline to pale olivaceous, guttulate, cylindrical to fusiform, truncate at the base, 6.5–22.1 × 1.3–3.2 μm ($\bar{x}$ = 13.8 × 2.5 μm, *n* = 20) (Figure 17F–L).

Culture characters: Colony on MEA growing slowly, attaining 21-mm-diam. after four weeks at 25 °C. Colony surface arising centrally, velvety with short pale drab-gray to smoke gray aerial hyphae, dry, lobate margin; reverse benzo brown (Figure 17A,B). Minimum 4 °C, optimum at 20–25 °C, and maximum 29 °C.

Additional specimen examined: China: Tibet: Nyingchi, Qinghai-Tibet Plateau, 29°45′ N, 94°45′ E, 3448 m a.s.l. from rock, 2 August 2013, Meichun Xiang, (living culture CGMCC 3.17345) and ibid., (living culture CGMCC 3.17329).

Notes: The new species is phylogenically closed to *C. parmeliae* and *C. pseudocarrionii* (Figure 4). However, conidia of the new species are smaller and slender than that of those two species, while the conidia of *C. pseudocarrionii* are ellipsoid to fusiform; 5.5–9.5 × 2–4.5 μm compared to those of *C. parmeliae* are subspherical to ellipsoidal, 0 (-1)-septate, 7–13 × 4–6 μm [67,68].

*Cladophialophora tengchongensis* W. Sun, L. Su, M.C. Xiang and X.Z. Liu, sp. nov.

**Figure 18:**
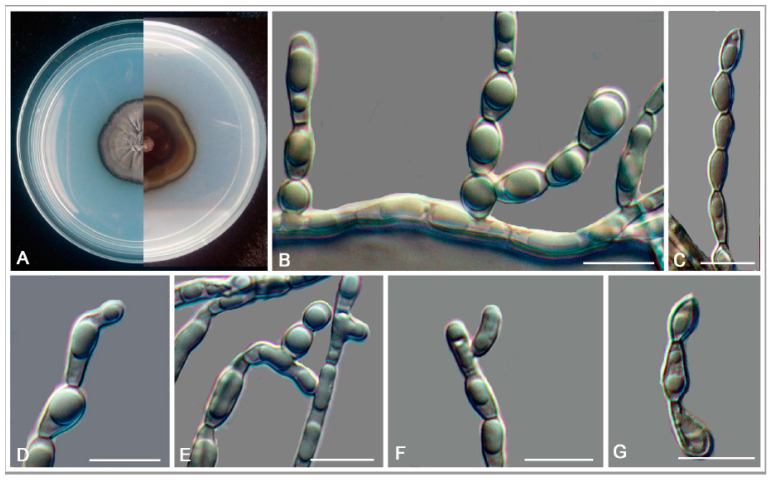
*Cladophialophora tengchongensis* (CGMCC 3.15201). (**A**) Forward (left) and reverse (right) of colony on MEA. (**B**–**G**) Conidiophore and conidial chains. Scale bars: (**B**–**G**) = 10 µm.

MycoBank MB 824206. (Figure 18)

Etymology: *tengchongensis* (Lat., masc. adj.), referring to the type locality.

Type: China: Yunnan Province: Tengchong, Tengchong Volcano National Geopark, 25°13′ N, 98°30′ E, 2000 m a.s.l., from rock, 28 April 2011, Lei Su, (HMAS 265607 (dried culture)–holotype and CGMCC 3.15201–ex-type culture).

Description: Hyphae smooth and thin-walled, septate, hyaline to pale olivaceous, composed by cylindrical to elongate cells, guttulate, branched, 1.9–4.0-μm-wide. Yeast cells absent. Conidiophores lateral or terminal, pale olivaceous, smooth and thin-walled, composed of ellipsoidal, fusiform, or subcylindrical cells (Figure 18B–E,G). Conidia one-celled, forming long branched chains, hyaline to pale olivaceous, ellipsoid to fusiform, 4.2–10.6 × 3.1–5.5 μm ($\bar{x}$ = 6.6 × 4.8 μm, *n* = 20) (Figure 18F).

Culture characters: Colony on MEA growing slowly, attaining 28-mm-diam., after four weeks at 25 °C. Colony surface raised centrally, pale gray to deep-olivaceous gray, flat and glossy near the periphery, zonate, with margin entire; reverse olive-brown to sepia (Figure 18A). Mycelium growing into the agar. No diffusible pigment produced. Minimum 4 °C, optimum at 20–25 °C, and maximum 28 °C.

Distribution: Tengchong (Yunnan Province, China).

Additional specimen examined: China: Yunnan Province: Tengchong, Tengchong Volcano National Geopark, 25°13′ N, 98°30′ E, 2000 m a.s.l., from rock, 28 April 2011, Lei Su, living culture CGMCC 3.15204.

Notes: *C. tengchongensis* is phylogenically close to *C. chaetospira* (Figure 4). However, those two species can be easily distinguished by conidial morphology; conidia of *C. tengchongensis* are one-celled and measure 4.2–10.6 × 3.1–5.5 μm; those of *C. chaetospira* are 1(-3)-septate and measure 25–30 × 3–4 μm [69].

*Exophiala cinerea* W. Sun, M.C. Xiang and X.Z. Liu, sp. nov.

**Figure 19:**
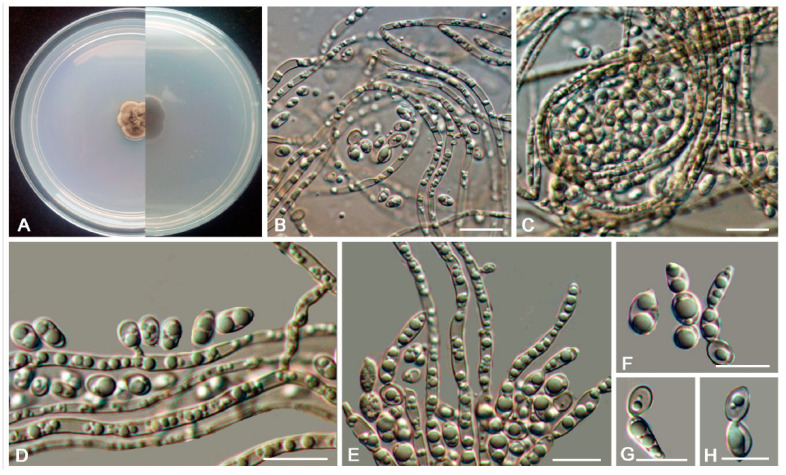
*Exophiala cinerea* (CGMCC 3.18778). (**A**) Forward (left) and reverse (right) of colony on MEA. (**B**,**C**) Spirally twisted hyphae. (**D**) Conidia alongside hyphae. (**E**) Conidial apparatus with conidia. (**F**–**H**) Conidia. Scale bars: (**B**–**H**) = 10 µm.

MycoBank MB 824205 (Figure 19)

Etymology: *cinerea* (Lat., fem. adj.), referring to the gray color of the colony.

Type: China: Guizhou Province: Leishan County, Leigongshan National Nature Reserve, 26°23′ N, 108°12′ E, 2152 m a.s.l., from rock, 13 October 2014, Meichun Xiang, (HMAS 247722 (dried culture)–holotype and CGMCC 3.18778–ex-type culture).

Description: Hyphae poorly branched, spirally twisted hyphae present, smooth and thin-walled, septate, guttulate, hyaline to light olive-grey, 1.0–4.1-μm-diam. (Figure 19B,C). Yeast cells absent. Conidiogenous cells, ellipsoidal-shaped, ovoidal to elongate, intercalary or terminal (Figure 19E). Conidia, one-celled, surface smooth, thin-walled, arising alongside the hyphae or at the apex. Solitary, cylindrical, or truncate at the base, 3.6–8.8 × 2.7–5.4 μm ($\bar{x}$ = 5.6 × 3.7 μm, *n* = 20) (Figure 19D,F–H).

Culture characters: Colony on MEA growing slowly, attaining 15-mm-diam. after four weeks at 25 °C. Colony surface olivaceous grey to black, raised centrally, flat and glossy near the periphery, velvety with grayish-brown short aerial hyphae and irregularly lobate margin; reverse grey black (Figure 19A). No diffusible pigment produced. Minimum 4 °C, optimum at 20–25 °C, and maximum 28 °C.

Additional specimen examined: China: Guizhou Province: Leishan County, Leigongshan National Nature Reserve, 26°23′ N, 108°12′ E, 2152 m a.s.l., from rock, 13 October 2014, Meichun Xiang, living culture CGMCC 3.18779.

Notes: *E. cinerea* forms a sister clade of *E. ellipsoidea* with 99% ML and 1.00 Bayesian support (Figure 5). *E. cinerea* is morphologically similar to *E. ellipsoidea*. However, *E. cinerea* has longer conidia (3.6–8.8 × 2.7–5.4 μm vs. 2.1–6.4 × 1.1–4.0 μm) than that of *E. ellipsoidea*.

*Exophiala clavispora* W. Sun, M.C. Xiang and X.Z. Liu, sp. nov.

**Figure 20:**
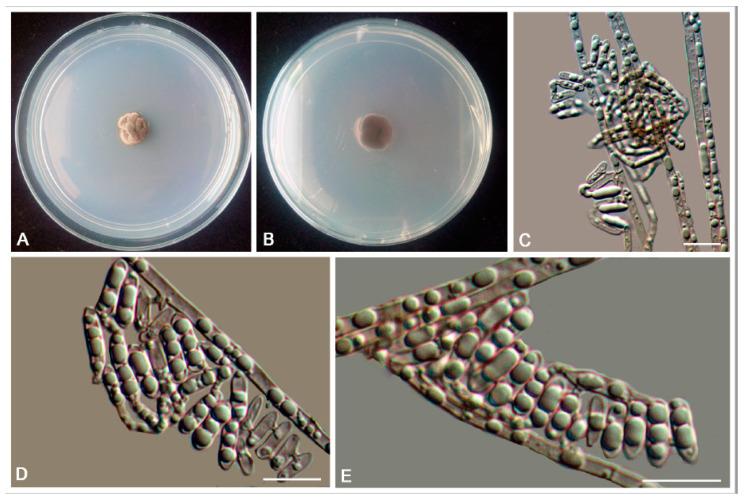
*Exophiala clavispora* (CGMCC 3.17517). (**A**,**B**) Forward and reverse of colony on MEA. (**C**) Spirally twisted conidial chains. (**D**,**E**) Conidia alongside hyphae. Scale bars: (**C**–**E**) = 10 µm.

MycoBank MB 824207 (Figure 20)

Etymology: *clavispora* (Lat., fem. adj.), referring to the clavate shape of the conidia.

Type: China: Tibet: Nyingchi, Qinghai-Tibet Plateau, 20°45′ N, 94°45′ E, 3448 m a.s.l., from rock, 2 August 2013, Meichun Xiang, (HMAS245389 (dried culture)–holotype and CGMCC 3.17517–ex-type culture).

Description: Hyphae composed by cylindrical to elongate cells, hyaline, guttulate, septate, 1.1–3.2-μm-wide, spirally twisted hyphae frequently observed (Figure 20C). Yeast cells absent. Conidiogenous cells ovoidal to elongate, olivaceous grey, 6.2–10.1-μm-long (Figure 20E). Conidia arising alongside the hyphae or at the apex, cylindrical to clavate, truncate at the base, 5.9–11.9 × 1.8–3.7 μm ($\bar{x}$ = 8.1 × 2.3 μm, *n* = 20) (Figure 20D,E).

Culture characters: Colony on MEA growing slowly, attaining 12-mm-diam. after four weeks at 25 °C. Colony surface cerebriform, arise centrally, velvety with grayish short aerial hyphae and lobate margin; reverse deep olivaceous gray (Figure 20A,B). Mycelium submerged into the agar. No diffusible pigment produced. Minimum 4 °C, optimum at 20–25 °C, and maximum 28 °C.

Additional specimen examined: China: Tibet: Nyingchi, Qinghai-Tibet Plateau, 20°45′ N, 94°45′ E, 3448 m a.s.l., from rock, 2 August 2013, Meichun Xiang, living culture CGMCC 3.17512 and ibid., living culture CGMCC 3.17513.

Diagnosis: *E. clavispora* morphologically resembles *E. bonariae* Isola and Zucconi [20], but *E. bonariae* has shorter conidia (5.5–6.5 × 2.2–2.8 μm).

*Exophiala ellipsoidea* W. Sun, L. Su, M.C. Xiang and X.Z. Liu, sp. nov.

**Figure 21:**
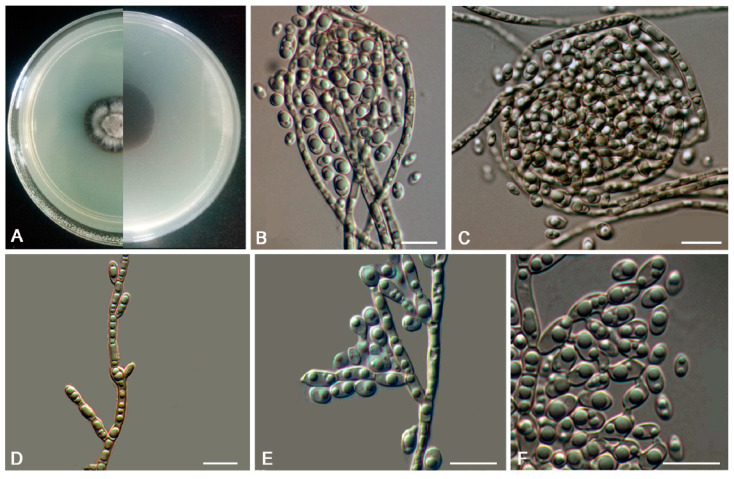
*Exophiala ellipsoidea* (CGMCC 3.17348). (**A**) Forward (left) and reverse (right) of colony on MEA. (**B**,**C**) Spirally twisted hyphae. (**D**–**F**) Conidiophores with branched conidial chains. Scale bars: (**B**–**F**) = 10 µm.

MycoBank MB 824208 (Figure 21)

Etymology: *ellipsoidea* (Lat., fem. adj.), referring to the ellipsoidal shape of conidia.

Type: China: Hebei Province: Zhangjiakou City, 40°23′ N, 114°51′ E, 1011 m a.s.l., from rock, 24 October 2013, Lei Su, (HMAS245364 (dried culture)–holotype and CGMCC 3.17348–ex-type culture).

Description: Hyphae septate, hyaline, spirally twisted hyphae frequently observed (Figure 21B,C). Yeast cells absent. Conidiogenous cells, ovoid to elongate, pale brown, 4.7–8.6-μm-long (Figure 21D–F). Conidia, one-celled, thin and smooth-walled, hyaline or pale olivaceous, broadly ellipsoidal to cylindrical with truncate hilum, 2.1–6.4 × 1.1–4.0 μm ($\bar{x}$ = 5.2 × 3.1 μm, *n* = 20) (Figure 21E,F).

Culture characters: Colony on MEA growing slowly, attaining 23-mm-diam. after four weeks at 25 °C. Colony surface velvety with pale olivaceous aerial mycelia at the center with a wide well-defined darker margin; reverse olivaceous black (Figure 21A). No diffusible pigment produced. Minimum 4 °C, optimum at 20–25 °C, and maximum 33 °C.

Additional specimen examined: China: Hebei Province: Zhangjiakou City, 40°23′ N, 114°51′ E, 1011 m a.s.l., from rock, 24 October 2013, Lei Su, living culture CGMCC 3.17521 and ibid., living culture CGMCC 3.17522.

Notes: Morphologically, *E. ellipsoidea* resembles *E. campbellii* in oval to ellipsoid conidia, but the conidia of *E. ellipsoidea* (2.1–6.4 × 1.1–4.0 μm ($\bar{x}$ = 5.2 × 3.1 μm, *n* = 20)) are longer than that of *E. campbellii* (3.5 to 5.0 by 2.0 to 3.0 μm) [70].

*Exophiala nagquensis* W. Sun, L. Su, M.C. Xiang and X.Z. Liu, sp. nov.

**Figure 22:**
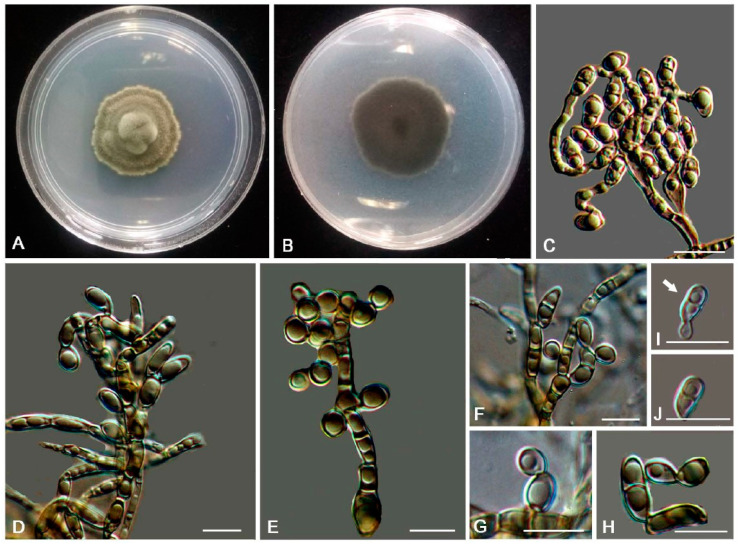
*Exophiala nagquensis* (CGMCC 3.17333). (**A**,**B**) Forward and reverse of colony on MEA. (**C**–**F**) Conidiophores with conidiogenous cells. (**G**–**J**) Conidia and budding cells. Scale bars: (**C**–**J**) = 10 µm.

MycoBank MB 824211 (Figure 22)

Etymology: *nagquensis* (Lat., masc. adj.), referring to the type locality.

Type: China: Tibet: Nagqu, 30°19′ N, 90°40′ E, 4591 m a.s.l., from rock, 4 August 2013, Meichun Xiang, (HMAS 245344 (dried culture)–holotype and CGMCC 3.17333–ex-type culture).

Description: Hyphae poorly branched, smooth-walled, septate, light olivaceous brown, 2.0–3.1-μm-diam. Budding cells consisting of subspherical cells occasionally observed (Figure 22I,H). Conidiogenous cells arising from undifferentiated hyphae, terminal or intercalary, brown, cylindrical, poorly differentiated, sometimes with sympodial conidiogenesis (Figure 22C–F). Conidia 0-1-septate, hyaline to pyrite yellow, broadly ellipsoid, obovoid, and global 4.8–10.4 × 2.6–5.0 μm ($\bar{x}$ = 7.8 × 3.3 μm, *n* = 20) (Figure 22G,J).

Culture characters: Colony on MEA growing slowly, attaining 34-mm-diam. after four weeks at 25 °C. Colony surface velvety with olivaceous grey short aerial hyphae and margin irregular, reverse olivaceous black (Figure 22A,B). No diffusible pigment produced. Minimum 4 °C, optimum at 20–25 °C, and maximum 28 °C.

Additional specimen examined: China: Tibet: Nangqu, 30°19′ N, 90°40′ E, 4591 m a.s.l., from rock, 4 August 2013, Meichun Xiang, living culture CGMCC 3.17284 and ibid., living culture CGMCC 3.17334.

Notes: *E. nagquensis* forms a sister clade to *E. brunnea* (CBS 587.66), with 96% ML and 1.00 Bayesian support (Figure 5). However, they can be distinguished by conidial morphology, and *E. brunnea* has narrower conidia (2–3 μm vs. 2.6–5.0 μm) compared with the new species. In the meanwhile, the budding cells are absent for *E. brunnea* [36] but present for the new species.

## 4. Discussion

The numbers of genera and species of RIF continues to expand [11,16,71,72]. Nonetheless, rock surfaces remain an underexplored habitat for fungi. The application of phylogenetic analysis to classify strains and delimit new genera and species has led to a significant expansion of new taxa in Dothidomycetes [19,21,73,74]. However, to our knowledge, RIF in the Eurotiomycetes have been poorly investigated [11,75]. During our preliminary classification of Chinese RIF using ITS barcoding, a number of strains were assignable to novel lineages in Chaetothyriales. In this study, we examined 31 RIF isolates affiliated in Trichomeriaceae. As a result, we established a new genus and 11 new species. The richness and broad distribution of RIF in Trichomeriaceae suggests that the RIF are the dominant and ubiquitous component of the rock microbiota. The overwhelming number of new species discovery indicates higher species than that previously estimated, and more investigation in extensive niches should likely reveal more new taxa.

*Exophiala* and *Cladophialophora* are two large and frequently encountered genera in Herpotrihiellaceae, but their taxonomy are problematic because of their less morphological characteristics. Traditionally, the conidia appear to be highly taxonomically informative at the species level of *Exophiala* and are widely used for the taxonomy. However, along with increased discovery of the new taxa, species delimitation in the genus *Exophiala* became difficult, because the conidial sizes frequently overlapped among morphologically similar but phylogenically distinct species, weakening the traditional morphological species concept. For instance, conidial sizes of *E. cinerea* (3.6–8.8 × 2.7–5.4 μm) and *E. nagquensis* (4.8–10.4 × 2.6–5.0 μm) are similar, but they are phylogenetically distinct (Figure 5). Therefore, the taxonomy of *Exophiala* should consider both morphological characteristics and phylogenetic relationships rather than relying on only one type of characters.

The species of *Cladophialophora* and *Exophiala* have very diverse habitats, such as plants, fruit juices, shower rooms, seawater, sports drinks, arable soil, wood pulp, oil sludge, and the decaying shell of babassu coconut [36,37,40,68,69,76], and have also been reported as opportunistic pathogens on the superficial skin or internal organs in humans and animals [35,37,38,44,46,70,77,78]. In this study, we isolated *Cladophialophora* and *Exophiala* mostly from rocks, indicating that rock is an important habitat for these species. Moreover, as a rock surface is normally dry with high solar irradiation, temperatures, and osmotic stress, the condition of the human body is in high temperature, rich in nutrients and high osmotic pressure. It is possible that species of *Cladophialophora* and *Exophiala* were isolated from and adapted to rocks.

Further extensive samplings and investigation of those fungi are necessary to generate a more complete knowledge about their biodiversity, distribution, habitats, and the adaptation mechanism to the stressing environment. Classification and species identification by DNA sequencing [79,80,81] and the multi-locus analysis [3,21] have overcome the limitations imposed by the morphological classification of organisms with cryptic vegetative features and reduced the capacity for sporulation in vitro. Rapid DNA sequencing and phylogenetic classification provide a framework for future inventories and for linking strains of species that occupy multiple habitats, e.g., natural rocks, manmade objects, plant surfaces, or animals. Although many studies have employed different loci combinations, the combination of two loci, e.g., ITS and nucLSU, have readily distinguished new species within the Trichomeriaceae and Herportichiellaceae families [21,73]. A three-gene analysis based on combined ITS, nucSSU, and mtSSU was conducted for the RIF species position within the order Capnodiales [21]. Three genes, including ITS, nucSSU, and mtSSU, and five loci of a combined nucLSU, nucSSU, mtSSU, RPB1, and RPB2 phylogenetic tree were established and compared in the statement of the phylogenetic placement of RIF within Dothideomyceta [21]. Other multi-gene phylogenetic analyses, such as four loci (nucLSU, nucSSU, mtSSU, and RPB1) and even six loci (ITS, nucLSU, nucSSU, mtSSU, TUB2, and RPB2) were also conducted in the species delimitation of Coniosporium [82] and Rupestriomyces and Spissiomyces [47]. Based on previous studies and our research experiences, a strategy for RIF identification and taxonomy can be proposed. At the outset, when new strains are accumulated, ITS sequences undergo preliminary screening as to determine if the isolates belong to major lineages of RIF or if they represent other environmental fungi that are isolated incidentally from rock surfaces. At a second step, the ITS and nucLSU combined sequences should be applied to assign the fungal isolates to order, family, and genus [11,28]. Finally, a combination of four loci sequences are selected from among the ITS, nucLSU, nucSSU, mtSSU, TUB, RPB1, and/or RPB2, etc. for species delimitation. To calibrate the phylogenetic analyses and validate the segregation of the new species proposed here, the data were analyzed by the poisson tree processes (PTP) server: a Bayesian implementation of the PTP model for species delimitation, and the results well-supported species delimitation and the taxonomic strategy of RIF (Figures S1–S4).

Although RIF can be readily isolated from well-vegetated and humid habitats, the ancestral RIF are hypothesized to originate during periods of dry climates in the late Devonian and middle-Triassic, when the paleoclimate subjected fungi to harsh environment stresses and were selected for colonizing rock surfaces in nutrient-depleted habitats [25]. Meristematic growth ensures an optimal volume/surface ratio and minimizes environmental exposure [83]. Highly melanized cell walls and the biosynthesis of mycosporines and mycosporine-like amino acids protect RIF against UV radiation [84,85]. Endospores and reproduction by vegetative fragmentation reduce resource investments in reproduction [25]. Loss of a sexual stage can simplify life cycles and enable dispersal during brief periods of optimal environmental conditions [2]. RIF produce a minimal array of metabolites, only those for essential survival—for example, extracellular polymeric substances [79], and, in general, they are poor antibiotic producers [86]. Presently, the origin, evolution, and mechanisms of stress adaptation of RIF are beginning to be understood comprehensively by a combination of genome, transcriptome, and proteome studies [26,87,88]. Although transcriptomic and metabolic analyses may explain details about their differential gene expressions and metabolic activity variations under stress, in order to reveal the survival strategies of RIF, further interesting researches are needed—for instance, how rapidly the transcriptional regulation initiated responding to fluctuating hydration conditions and how to resume metabolic activities after long periods of metabolic suspense undergoing extremes [88].

## Figures and Tables

**Table 1:** List of primers and annealing temperatures for amplification of the target loci.

Locus	Primer	Annealing Temperature (°C)	Orientation	Literature
ACT	Act1	52	Forward	[51]
ACT	Act5ra	52	Reverse	[51]
ITS	ITS5	54	Forward	[52]
ITS	ITS4	54	Reverse	[52]
mtSSU	mrSSU1	55	Forward	[53]
mtSSU	mrSSU3R	55	Reverse	[53]
nucLSU	LROR	52	Forward	[54]
nucLSU	LR5	52	Reverse	[55]
RPB1	RPB1-Af	51	Forward	[56]
RPB1	RPB1-Cr	51	Reverse	[56]
SSU	NS1	52	Forward	[52]
SSU	NS4	52	Reverse	[52]
TEF	EF1-728F	57	Forward	[57]
TEF	EF1-986R	57	Reverse	[57]
TUB	Bt2a	52	Forward	[58]
TUB	Bt2b	52	Reverse	[58]
TUB	T1	52	Forward	[59]
TUB	T22	52	Reverse	[59]

## Supplementary Materials

The following are available online at https://www.mdpi.com/2309-608X/6/4/187/s1. Table S1: GenBank accession numbers of taxa used in phylogenetic analyses. Figure S1: Phylogenetic tree generated by PTP server analysis using the combined ITS and LSU sequences to calibrate the generic positions of RIF affiliated in Chaetothyriales. Figure S2: Phylogenetic tree generated by PTP server using combined sequences of ITS, nucLSU, mtSSU, RPB1, and TUB loci to validate the new species in the family Trichomeriaceae. Figure S3: Phylogenetic tree generated by PTP server using combined sequences of ITS, SSU, TEF, TUB and ACT loci to validate the new species in *Exophiala*. Figure S4: Phylogenetic tree generated by PTP server analysis using combined sequences of ITS, SSU, TEF, TUB and nucLSU to validate the new species in *Cladophialophora*.
