# Supplementary material for: Unveiling the Hidden Diversity of Rock-Inhabiting Fungi: Chaetothyriales from China
Source: J Fungi (Basel). 2020 Sep 24;6(4):187. doi: 10.3390/jof6040187 (PMC7711927; doi:10.3390/jof6040187)

Supplementary Figures

Figure 1. Phylogenetic tree generated by PTP server analysis using the combined ITS and LSU sequences to calibrate the generic positions of RIF affiliated in Chaetothyriales


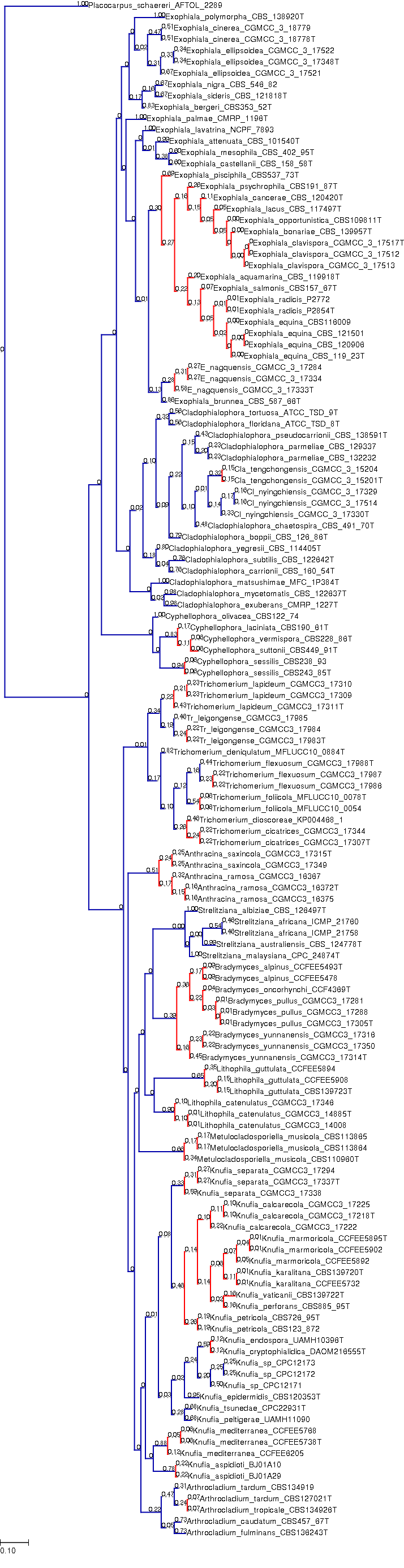


Figure 2. Phylogenetic tree generated by PTP server using combined sequences of ITS, nucLSU, mtSSU, RPB1, and TUB loci to validate the new species in the family Trichomeriaceae.
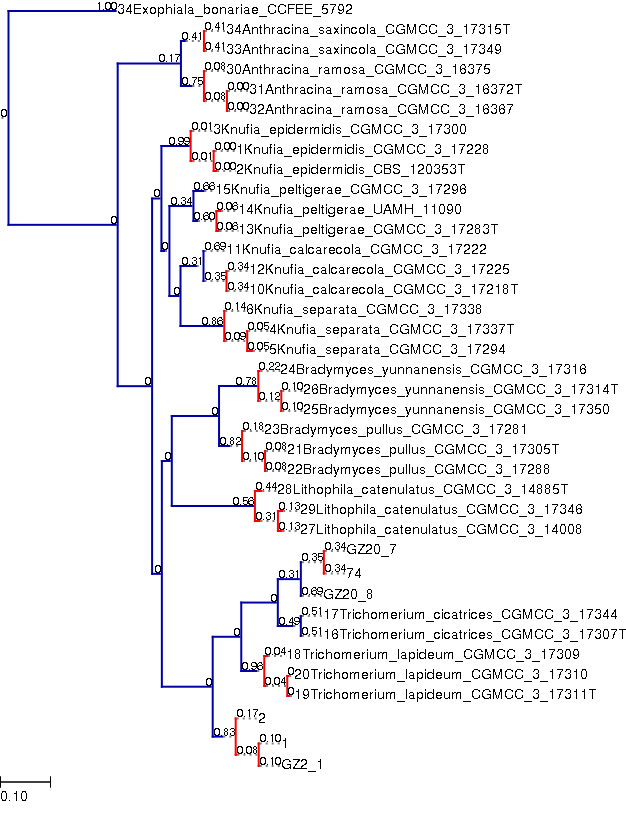


Figure 3. Phylogenetic tree generated by PTP server using combined sequences of ITS, SSU, TEF, TUB and ACT loci to validate the new species in *Exophiala*.


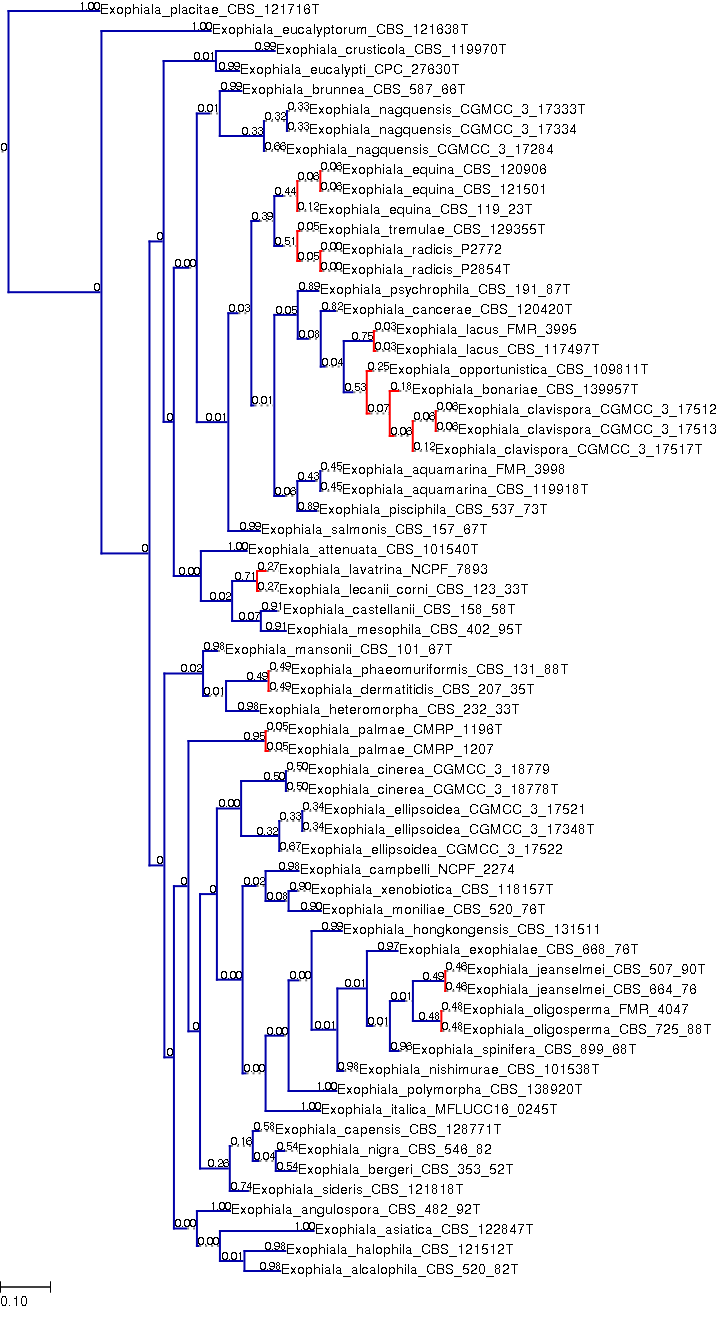


Figure 4. Phylogenetic tree generated by PTP server analysis using combined sequences of ITS, SSU, TEF, TUB and nucLSU to validate the new species in *Cladophialophora*.


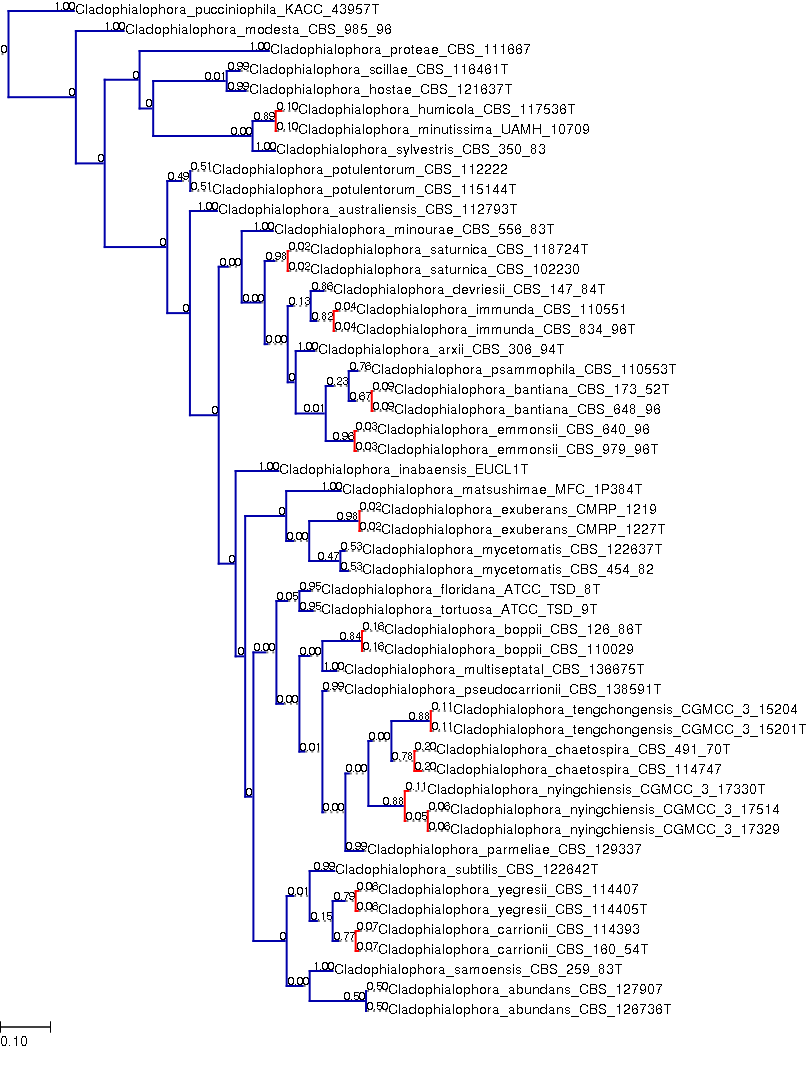

Supplement: Supplementary file 1 [file jof-06-00187-s001.zip › Supplementary table and figures/Supplementary Figures.docx]
