# Supplementary material for: Unveiling the Hidden Diversity of Rock-Inhabiting Fungi: Chaetothyriales from China
Source: J Fungi (Basel). 2020 Sep 24;6(4):187. doi: 10.3390/jof6040187 (PMC7711927; doi:10.3390/jof6040187)
Supplement: Supplementary file 1 [file jof-06-00187-s001.zip › Supplementary table and figures/Table S1..doc]

**Table S1.** GenBank accession numbers of taxa used in phylogenetic analyses.

Taxon, Strains no., Source, Locality (county/province/country), GenBank accession number (the order of loci used for the phylogenetic analysis is ITS, LSU, SSU, mtSSU, RPB1, TUB, TEF and ACT). The new isolates and generated sequences in this study are shown in bold. A dash (“-”) indicates missing data.

***Anthracina ramosa***, CGMCC 3.16372T, Dolomite, Henan, China, **KP174844**, **KP174923**, **KP174883**, **KP174984**, **KP226532**, **KP226558**, -, -; ***Anthracina ramosa***, CGMCC 3.16375, Rock, Ganshu, China, **KP174845**, **KP174925**, **KP174882**, **KP174985**, **KP226533**, **KP226557**, -, -; ***Anthracina ramosa***, CGMCC 3.16367, Dolomite, Henan, China, **KP174846**, **KP174922**, **KP174881**, **KP174986**, **KP226535**, **KP226556**, -, -; ***Anthracina saxincola***, CGMCC 3.17349, Rock, Yunnan, China, **KP174842**, **KP174921**, **KP174879**, **KP174983**, -, **KP226554**, -, -; ***Anthracina saxincola***, CGMCC 3.17315T, Rock, Yunnan, China, **KP174843**, **KP174924**, **KP174880**, **KP174987**, -, **KP226555**, -, -; *Arthrocladium caudatum*, CBS 457.67T, Leaf litter *Acacia karroo*, South Africa, KT337438, KT337443, -, -, -, -, -, -; *Arthrocladium fulminans*, CBS 136243T, Disseminated infection human, Africa, KT337439, KT337444, -, -, -, -, -, -; *Arthrocladium tardum*, CBS 127021T, Shell of coconut Babassu palm, Brazil, KT337441, KT337446, -, -, -, -, -, -; *Arthrocladium tardum*, CBS 134919, Petalomyrmex phylax ant domatia in Leonardoxa africana, Cameroon, KT337442, KT337447, -, -, -, -, -, -; *Arthrocladium tropicale*, CBS 134926T, Petalomyrmex phylax ant domatia in Leonardoxa africana, Cameroon, KT337440, KT337445, -, -, -, -, -, -; *Bradymyces alpinus*, CCFEE5493T, Rock, Alps, Italy, HG793052, GU250396, -, -, -, -, -, -; *Bradymyces alpinus*, CCFEE 5478, Rock, Alps, Italy, HG974249, -, -, -, -, -, -, -; *Bradymyces oncorhynchi*, CCF 4369T, Spleen of *Oncorhynchus mykiss*, Czech Republic, HG426062, HG426063, -, -, -, -, -, -; ***Bradymyces pullus***, CGMCC 3.17305T, Rock, Tibet, China, **KP174870**, **KP174929**, **KP174905**, **KP174956**, **KP226520**, **KP226550**, -, -; ***Bradymyces pullus***, CGMCC 3.17288, Rock, Tibet, China, **KP174868**, **KP174950**, **KP174901**, **KP174958**, **KP226521**, **KP226548**, -, -; ***Bradymyces pullus***, CGMCC 3.17281, Rock, Tibet, China, **KP174869**, **KP174949**, **KP174903**, **KP174957**, -, **KP226549**, -, -; ***Bradymyces yunnanensis***, CGMCC 3.17350, Rock, Yunnan, China, **KP174865**, **KP174926**, **KP174906**, **KP174955**, **KP226536**, **KP226545**, -, -; ***Bradymyces yunnanensis***, CGMCC 3.17316, Rock, Yunnan, China, **KP174867**, **KP174927**, **KP174907**, **KP174953**, -, **KP226547**, -, -; ***Bradymyces yunnanensis***, CGMCC 3.17314T, Rock, Yunnan, China, **KP174866**, **KP174928**, **KP174904**, **KP174954**, -, KP226546, -, -; *Cladophialophora abundans*, CBS 126736T, Thorn of Japecanga plant, Bacabeira city, Maranhao, KC776592, KC812100, -, -, -, -, -, -; *Cladophialophora abundans*, CBS 127907, Soil from mangrove environment, Bahia state, Acupi, KC776597, -, -, -, -, -, -, -; *Cladophialophora arxii*, CBS 306.94T, man tracheal abscess, Germany, EU103986, KX822320, AJ232948, -, -, -, EU140593, -; *Cladophialophora australiensis*, CBS 112793T, Sport drink, Australia, EU035402, -, KX822275, -, -, -, -, -; *Cladophialophora bantiana*, CBS 173.52T, Human, brain abscess, USA, EU103989, KF155189, AY554284, -, -, -, EU140585, -; *Cladophialophora bantiana*, CBS 648.96, Liver, Dog, -, EU103993, -, -, -, -, -, EU140587, -; *Cladophialophora boppii*, CBS 110029, Scales of face, male, Netherlands, Dordrecht, EU103998, -, -, -, -, -, EU140597, -; *Cladophialophora boppii*, CBS 126.86T, man skin lesion, on limb, Brazil, EU103997, FJ358233, FJ358301, -, -, -, EU140596, -; *Cladophialophora carrionii*, CBS 114393, Chromoblastomycosis hand, male 72, Falcon State, Venezuela, KF928452, KF928516, -, -, -, EU137151, EU137212, -; *Cladophialophora carrionii*, CBS 160.54T, man case of chromoblastomycosis, Australia, EU137266, FJ358234, FJ358302, -, -, EU137201, EU137210, -; *Cladophialophora chaetospira*, CBS 114747, *Phyllostachys bambusoides*, China, EU035403, KF928514, -, -, -, KF928578, -, -; *Cladophialophora chaetospira*, CBS 491.70T, Roots of *Picea abies*, Denmark, EU035405, EU035405, KX822276, -, -, -, -, -; *Cladophialophora devriesii*, CBS 147.84T, Human, disseminated infection, USA, Grand Cayman Islands, EU103985, KC809989, KF155192, -, -, -, EU140595, -; *Cladophialophora emmonsii*, CBS 640.96, Cat, subcutaneous lesion, -, EU103995, KC809995, -, -, -, -, EU140584, -; *Cladophialophora emmonsii*, CBS 979.96T, man phaeohyphomycosis, subcutaneous lesion right forearm, Virginia, EU103996, -, -, -, -, -, EU140583, -; *Cladophialophora exuberans*, CMRP1219, Orbignya phalerata from decaying shell of babassu coconut, Brazil, KY680430, KY570930, -, -, -, KY689827, -, -; *Cladophialophora exuberans*, CMRP1227T, Orbignya phalerata from decaying shell of babassu coconut, Brazil, KY680429, KY570931, -, -, -, KY689826, -, -; *Cladophialophora floridana*, ATCC TSD-8T, Sclerotium of Cenococcum geophilum, Florida, AB986343, AB986343, -, -, -, -, -, -; *Cladophialophora hostae*, CBS 121637T, *Hosta plantaginea*, Korea, KX822478, -, KX822277, -, -, -, -, -; *Cladophialophora humicola*, CBS 117536T, Soil, Germany, EU035408, KC809987, KJ636038, -, -, -, -, -; *Cladophialophora immunda*, CBS 110551, Soil, Netherlands, Apeldoom, AY857510, -, -, -, -, EU137207, EU137261, -; *Cladophialophora immunda*, CBS 834.96T, Sub-cutaneous phaeohyphomycosis, male, U.S.A., Georgia, Atlanta, EU137318, KC809990, KF155194, -, -, EU137203, EU137257, -; *Cladophialophora inabaensis*, EUCL1T, Secondary forest soil, Japan, LC128795, -, -, -, -, -, -, -; *Cladophialophora matsushimae*, MFC-1P384T, Decaying petiole of palma, Japan, FN549916, FN400758, -, -, -, -, -, -; *Cladophialophora minourae*, CBS 556.83T, Decaying wood, Japan, AY251087, FJ358235, FJ358303, -, -, -, EU140598, -; *Cladophialophora minutissima*, UAMH 10709T, *Polytrichum juniperinum*, Canada, NR_137550, -, EF016370, -, -, -, -, -; *Cladophialophora modesta*, CBS 985.96, man brain, USA, KF928421, KF928485, AJ232953, -, -, KF928549, -, -; *Cladophialophora multiseptata*, CBS 136675T, Soil, Spain, HG003668, HG003671, -, -, -, -, -, -; *Cladophialophora mycetomatis*, CBS 122637T, Eumycetoma, male, Mexico, Jicaltepec, FJ385276, KX822321, KX822278, -, -, -, -, -; *Cladophialophora mycetomatis*, CBS 454.82, Culture contaminant, Netherlands, EU137293, KC809991, KX822279, -, -, EU137176, EU137235, -; ***Cladophialophora nyingchiensis***, CGMCC 3.17329, Rock, Tibet, China, **MG012700**, **MG197825**, **MG012729**, -, -, **MG012748**, **MG012707**, -; ***Cladophialophora nyingchiensis***, CGMCC 3.17330T, Rock, Tibet, China, **MG012699**, **MG197824**, **MG012728**, -, -, **MG012747**, **MG012706**, -; ***Cladophialophora nyingchiensis***, CGMCC 3.17514, Rock, Tibet, China, **MG012701**, **MG197826**, **MG012730**, -, -, **MG012749**, **MG012708**, -; *Cladophialophora parmeliae*, CBS 129337, *Juniperus brevifolia*, Spain, JQ342180, JQ342182, -, -, -, -, -, -; *Cladophialophora parmeliae*, CBS 132232, *Hypotrachyna* sp., Spain, Azores, -, JX081671, -, -, -, -, -, -; *Cladophialophora potulentorum*, CBS 115144T, Apple juice, -, DQ008141, -, -, -, -, -, -, -; *Cladophialophora potulentorum*, CBS 112222, Sports drink, Australia, EU035409, -, KX822280, -, -, -, -, -; *Cladophialophora proteae*, CBS 111667T, *Protea cynaroides*, South Africa, EU035411, EU035411, KJ636039, -, -, -, -, -; *Cladophialophora psammophila*, CBS 110553T, Gasoline-polluted soil, Netherlands, AY857517, -, -, -, -, -, -, -; *Cladophialophora pseudocarrionii*, CBS 138591T(= FMR 12062), Soil, Spain, KU705827, KU705844, -, -, -, -, -, -; *Cladophialophora pucciniophila*, KACC 43957T, on rust telia of *Puccinia polygoni-amphibii* on *Persicaria fauriei*, South Korea, NR_137769, JF263534, -, -, -, -, -, -; *Cladophialophora samoensis*, CBS 259.83T, Chromoblastomycosis skin lesion, male, USA, EU137291, KC809992, KX822281, -, -, EU137174, EU137233, -; *Cladophialophora saturnica*, CBS 102230, Litter, vegetable cover/soil, Paraná, Curitiba, Brazil, AY857508, KC809993, KX822282, -, -, -, EU140600, -; *Cladophialophora saturnica*, CBS 118724T, Interdigital toe lesion, child, Paraná, Curitiba, Brazil, EU103984, -, -, -, -, -, EU140602, -; *Cladophialophora scillae*, CBS 116461T, *Scilla peruviana*, New Zealand, EU035412, -, KJ636040, -, -, -, -, -; *Cladophialophora subtilis*, CBS 122642T, Ice tea, Netherlands, Utrecht, FJ385273, KX822322, KX822283, -, -, -, -, -; *Cladophialophora sylvestris*, CBS 350.83, *Pinus sylvestris*, Netherlands, EU035413, -, KJ636041, -, -, -, -, -; ***Cladophialophora tengchongensis***, CGMCC3.15201T, Rock, Yunnan, China, **MG012702**, **MG197827**, **MG012731**, -, -, **MG012750**, **MG012709**, -; ***Cladophialophora tengchongensis***, CGMCC3.15204, Rock, Yunnan, China, **MG012703**, **MG197828**, **MG012732**, -, -, **MG012751**, **MG012710**, -; *Cladophialophora tortuosa*, ATCC TSD-9T, Sclerotium of *Cenococcum geophilum*, Florida, AB986424, AB986424, -, -, -, -, -, -; *Cladophialophora yegresii*, CBS 114405T, *Stenocereus griseus* asymptomatic plant, Venezuela, EU137322, KX822323, KX822284, -, -, EU137209, EU137262, -; *Cladophialophora yegresii*, CBS 114407, *Stenocereus griseus* asymptomatic plant, Venezuela, EU137324, -, -, -, -, -, EU137264, -; *Cyphellophora laciniata*, CBS 190.61T, Man skin, Switzerland, EU035416, FJ358239, -, -, -, -, -, -; *Cyphellophora olivacea*, CBS 122.74, Moist wallpaper, Germany, KC455247, KC455260, -, -, -, -, -, -; *Cyphellophora sessilis*, CBS 243.85T, Resin of *Picea abies*, Netherlands, EU514700, -, -, -, -, -, -, -; *Cyphellophora sessilis*, CBS 238.93, Styrene, Netherlands, AY857541, KC455264, -, -, -, -, -, -; *Cyphellophora suttoni*, CBS 449.91T, Dog’s ear subcutaneous lesion, USA, KC455243, KC455256, -, -, -, -, -, -; *Cyphellophora vermispora*, CBS 228.86T, Root of *Triticum aestivum*, Germany, KC455244, KC455257, -, -, -, -, -, -; *Exophiala alcalophila*, CBS 520.82T, Soil, Japan, JF747041, AF361051, JN856010, -, -, JN112423, JN128771, JN112379; *Exophiala angulospora*, CBS 482.92T, water from drinking well, Japan, JF747046, KF155190, JN856011, -, -, JN112426, JN128780, JN112383; *Exophiala aquamarina*, CBS 119918T, Skin of leafy sea dragon, USA, JF747054, -, JN856012, -, -, JN112434, -, JN112388; *Exophiala aquamarina*, FMR 3998, River sediments, Tarragona, Spain, KU705829, KU705846, -, -, -, -, -, -; *Exophiala asiatica*, CBS 122847T, Wound infection of tonsils, human, China, EU910265, -, -, -, -, -, -, -; *Exophiala attenuata*, CBS 101540T, Soil, Colombia, AF549446, -, -, -, -, -, -, -; *Exophiala bergeri*, CBS 353.52T, Skin infection, human, Canada, EF551462, FJ358240, FJ358308, -, -, EF551497, EF551524, EF551464; *Exophiala bonariae*, CBS 139957T = CCFEE 5792, Marble, Italy, JX681046, KR781083, -, -, -, -, -, -; *Exophiala brunnea*, CBS 587.66T, Litter of *Acacia karoo*, South Africa, JF747062, KX712342, JN856013, -, -, JN112442, JN128783, JN112393; *Exophiala campbellii*, NCPF 2274, Isolated from subcutaneous lesion (foot ganglion), Human, Germany, LT594703, LT594760, -, -, -, -, LT594739, -; *Exophiala cancerae*, CBS 120420T, Diseased mangrove crab, Brazil, JF747064, -, -, -, -, JN112444, JN128800, JN112394; *Exophiala capensis*, CBS 128771T, Leaf of *Phaenocoma prolifera* (L.) D. Don, Western Cape Province, South Africa, JF499841, -, -, -, -, -, -, -; *Exophiala castellanii*, CBS 158.58T, Human, Sri Lanka, JF747070, KF928522, JN856014, -, -, KF928586, JN128766, -; ***Exophiala cinerea***, CGMCC 3.18778T, Rock, Guizhou, China, **MG012695**, **MG197820**, **MG012724**, -, -, **MG012745**, **MG012704**, **MG012714**; ***Exophiala cinerea***, CGMCC 3.18779, Rock, Guizhou, China, **MG012696**, **MG197821**, **MG012725**, -, -, **MG012746**, **MG012705**, **MG012715**; ***Exophiala clavispora***, CGMCC 3.17512, Rock, Tibet, China, **KP347940**, **MG197829**, **MG012733**, -, -, **KP347931**, **KP347909**, **MG012712**; ***Exophiala clavispora***, CGMCC 3.17513, Rock, Tibet, China, **KP347943**, **MG197830**, **MG012734**, -, -, **KP347933**, **KP347910**, **KP347892**; ***Exophiala clavispora***, CGMCC 3.17517T, Rock, Tibet, China, **KP347942**, **KP347964**, **KP347967**, -, -, **KP347932**, **KP347911**, **KP347893**; *Exophiala crusticola*, CBS 119970T, Biological soil crust, Colorado Plateau, USA, AM048755, KF155180, KF155199, -, -, -, -, -; *Exophiala dermatitidis*, CBS 207.35T, Facial chromomycosis, Japan, AF050269, KJ930160, -, -, -, KF928572, -, -; ***Exophiala ellipsoidea***, CGMCC 3.17348T, Rock, Hebei, China, **KP347955**, **KP347956**, **KP347965**, -, -, **KP347921**, **KP347901**, **MG012713**; ***Exophiala ellipsoidea***, CGMCC 3.17521, Rock, Hebei, China, **KP347953**, **MG197832**, **MG012736**, -, -, **KP347920**, **KP347902**, **KP347885**; ***Exophiala ellipsoidea***, CGMCC 3.17522, Rock, Hebei, China, **KP347954**, **MG197831**, **MG012735**, -, -, **KP347919**, -, **KP347884**; *Exophiala equina*, CBS 119.23T, Subcutaneous infection of horse, Italy, JF747094, -, JN856017, -, -, JN112462, JN128814, JN112401; *Exophiala equina*, CBS 120906, Stool, USA, JF747093, -, -, -, -, JN112461, JN128813, JN112400; *Exophiala equina*, CBS 121501, Drinking water, Netherlands, JF747077, -, -, -, -, JN112453, JN128806, JN112398; *Exophiala equina*, CBS 116009, Lung tissue of Galapagos tortoise, zoo, Chicago, USA, JF747095, KF155181, -, -, -, -, -, -; *Exophiala eucalypti*, CPC 27630T = CBS 142069, on leaves of *Eucalyptus* sp., South Africa, KY173411, KY173502, -, -, -, -, -, -; *Exophiala eucalyptorum*, CBS 121638T = CPC 11261, on leaf litter of *Eucalyptus* sp., New Zealand, NR_132882, KC455258, KC455302, -, -, KC455228, -, -; *Exophiala exophialae*, CBS 668.76T, Straw in an armadillo burrow, Uruguay, AY156973, KX822326, KX822287, -, -, EF551499, EF551526, EF551466; *Exophiala heteromorpha*, CBS 232.33T, Wood pulp, Sweden, AY857524, -, -, -, -, -, -, -; *Exophiala hongkongensis*, CBS 131511, Big toe nail infection, human, Hong Kong, China, JN625231, -, -, -, -, JN625236, -, JN625241; *Exophiala italica*, MFLUCC16-0245T, *Cytisus scoparius* on dead branch, Italy, KY496744, KY496723, KY501114, -, -, -, KY514393, -; *Exophiala jeanselmei*, CBS 507.90T, Man, Uruguay, AY156963, FJ358242, FJ358310, -, -, EF551501, EF551530, -; *Exophiala jeanselmei*, CBS 664.76, Human, -, AF050271, -, -, -, -, -, -, -; *Exophiala lacus*, FMR 3995, River sediments, Ampuriabrava, Girona, Spain, KU705830, KU705847, -, -, -, -, -, -; *Exophiala lacus*, CBS 117497T, Lake water, North Holland, Netherlands, JF747110, -, -, -, -, -, JN128776, JN112407; *Exophiala lavatrina*, NCPF 7893, Domestic bathroom, United Kingdom, LT594696, LT594755, -, -, -, -, LT594729, - *Exophiala lecanii-corni*, CBS 123.33T, Lecanium corni, -, AY857528, FJ358243, FJ358311, -, -, -, -, -; *Exophiala mansonii*, CBS 101.67T, *Populus tremula* L., Sweden, AF050247, AY004338, X79318, -, -, -, -, -; *Exophiala mesophila*, CBS 402.95T, Silicon seal in shower room of a hospital, Hamburg, Germany, JF747111, KX712349, JN856016, -, -, JN112476, JN128761, -; *Exophiala moniliae*, CBS 520.76T, Twig of *Quercus* sp., St. Petersburg, Northwestern Federal District, Russia, KF881967, KJ930162, -, -, -, -, -, -; ***Exophiala nagquensis***, CGMCC 3.17284, Rock，Tibet, China, **KP347947**, **MG197838**, **MG012742**, -, -, **KP347922**, **KP347913**, **KP347894**; ***Exophiala nagquensis***, CGMCC 3.17333T, Rock, Tibet, China, **KP347948**, **KP347960**, **KP347970**, -, -, **KP347924**, **KP347914**, **KP347895**; ***Exophiala nagquensis***, CGMCC 3.17334, Rock, Tibet, China, **KP347949**, **MG197837**, **MG012741**, -, -, **KP347923**, **KP347915**, **KP347896**; *Exophiala nigra*, CBS 535.94T, Seawater, Russia, KY115191, KX712353, -, -, -, -, -, -; *Exophiala nishimurae*, CBS 101538T, Culture contaminant, Venezuela, AY163560, KX822327, KX822288, -, -, JX482552, EF551523, JX482553; *Exophiala oligosperma*, CBS 725.88T, Cerebral phaeohyphomycosis, Frankfurt-am-Main, Germany, AY163551, KF928486, FJ358313, -, -, EF551508, EF551534, EF551474; *Exophiala oligosperma*, FMR 4047, River sediments, Barcelona, Spain, KU705831, KU705848, -, -, -, -, -, -; *Exophiala opportunistica*, CBS 109811T, Drinking water, Germany, JF747123, KF928501, -, -, -, JN112486, JN128792, JN112408; *Exophiala palmae*, CMRP1196T, decaying shell of babassu coconut (*Orbignya phalerata*), Brazil, KY680434, KY570929, -, -, -, KY689829, -, -; *Exophiala palmae*, CMRP1207, decaying shell of babassu coconut (*Orbignya phalerata*), Brazil, KY680433, KY570928, -, -, -, KY689828, -, -; *Exophiala phaeomuriformis*, CBS 131.88T, Cutaneous mycosis of 48-year-old female patient, Japan, AJ244259, -, -, -, -, -, -, -; *Exophiala pisciphila*, CBS 537.73T, Catfish, USA, NR121269, AF361052, JN856018, -, -, JN112493, JN128788, JN112412; *Exophiala placitae*, CBS 121716T, Leaf of *Eucalyptus placita*, New South Wales, Australia, EU040215, -, -, -, -, -, -, -; *Exophiala polymorpha*, CBS 138920T, man, San Antonio, USA, KP070763, KP070764, -, -, -, -, -, -; *Exophiala psychrophila*, CBS 191.87T, Salmon in fish farm, Norway, JF747135, -, JN856019, -, -, JN112497, JN128798, -; *Exophiala radicis*, P2772, Root endophyte of *Microthlaspi erraticum*, Germany, KT099203, KT723447, KT723452, -, -, KT723462, KT723457, KT723442; *Exophiala radicis*, P2854T, Root endophyte of *Microthlaspi perfoliatum*, Germany, KT099204, KT723448, KT723453, -, -, KT723463, KT723458, KT723443; *Exophiala salmonis*, CBS 157.67T, Cerebral mycetoma of salmon, Canada, AF050274, AY213702, JN856020, -, -, JN112499, JN128747, JN112415; *Exophiala sideris*, CBS 121818T, soil polluted aromatic componds, Netherlands, HQ452311, -, HQ441174, -, -, HQ535833, HQ452336, -; *Exophiala spinifera*, CBS 899.68T, man nasal granuloma, USA, AY156976, -, -, -, -, EF551516, EF551541, EF551482; *Exophiala tremulae*, CBS 129355T, Populus tremuloides roots of apparently healthy 5-7-year-old sapling, Alberta, FJ665274, -, KT894147, -, -, KT894148, KT894149, KT894146; *Exophiala xenobiotica*, CBS 118157T, oil sludge, Venezuela, DQ182587, -, -, -, -, -, -, -; *Knufia aspidioti*, BJ01A29, *Aspidiotus* sp. (scale insect), China, KF589944, -, -, -, -, -, -, -; *Knufia aspidioti*, BJ01A10, *Aspidiotus* sp. (scale insect), China, KF589943, -, -, -, -, -, -, -; ***Knufia calcarecola***, CGMCC 3.17225, limestone, Beijing, China, **JQ354912**, **KP174912**, **KP174877**, **KP174968**, **KP226504**, -, -, -; ***Knufia calcarecola***, CGMCC 3.17222, limestone, Beijing, China, **KP174862**, **KP174914**, **KP174884**, **KP174966**, **KP226505**, -, -, -; ***Knufia calcarecola***, CGMCC 3.17218T, limestone, Beijing, China, **JQ354925**, **KP174915**, **KP174878**, **KP174967**, **KP226506**, -, -, -; *Knufia cryptophialidica*, DAOM 216555T, *Populus tremuloides*, Canada, JN040500, JN040500, -, -, -, -, -, -; *Knufia endospora*, UAMH 10396T, *Populus tremuloides*, Canada, JN040510, -, -, -, -, -, -, -; *Knufia epidermidis*, CBS 120353T, Foot infection, China, EU730589, FJ355954, -, -, -, -, -, -; *Knufia karalitana*, CBS 139720T = CCFEE 5656, Marble, Cathedral S. Maria, Cagliari, Italy, KP791781, KR781069, -, -, -, -, -, -; *Knufia karalitana*, CCFEE 5732, Pietraforte (sandstone), Boyl Palace, Cagliari, Italy, KP791782, KR781070, -, -, -, -, -, -; *Knufia marmoricola*, CCFEE 5892, Travertine, St Peter’s colonnade, Vatican City State, KP791778, KR781066, -, -, -, -, -, -; *Knufia marmoricola*, CBS 139726T = CCFEE 5895, Travertine, St Peter’s colonnade, Vatican City State, KP791775, KR781063, -, -, -, -, -, -; *Knufia marmoricola*, CCFEE 5902, Marble capital, CP, Vatican City State, KP791776, KR781064, -, -, -, -, -, -; *Knufia mediterranea*, CBS 139721T = CCFEE 5738, Marble, F. Warzee funerary monument, CB, Italy, KP791791, KR781078, -, -, -, -, -, -; *Knufia mediterranea*, CCFEE 5768, Marble, G. Todde funerary monument, CB, Italy, KP791792, KR781079, -, -, -, -, -, -; *Knufia mediterranea*, CCFEE 6205, Marble, F. Warzee funerary monument, CB, Italy, KP791794, KR781081, -, -, -, -, -, -; *Knufia peltigerae*, UAMH 11090, Thalli of *Peltigera rufescens*, Luxembourg, HQ709322, HQ613813, -, -, -, -, -, -; *Knufia perforans*, CBS 885.95T, Marble, Greece, AJ244230, FJ358237, -, -, -, -, -, -; *Knufia petricola*, CBS 123.872= A95, Marble statue, Messina, Italy, KC978734, FJ358269, -, -, -, -, -, -; *Knufia petricola*, CBS 726.95T, Marble statue, Messina, Italy, KC978746, KC978741, -, -, -, -, -, -; ***Knufia separata***, CGMCC 3.17294, Conglomerate, Tibet, China, **KP174855**, **KP174931**, **KP174874**, **KP174974**, **KP226514**, **KP226565**, -, -; ***Knufia separata***, CGMCC 3.17338, Conglomerate, Tibet, China, **KP174857**, **KP174933**, **KP174888**, **KP174972**, **KP226515**, **KP226563**, -, -; ***Knufia separata***, CGMCC 3.17337T, Conglomerate, Tibet, China, **KP174856**, **KP174932**, **KP174873**, **KP174973**, **KP226516**, **KP226564**, -, -; *Knufia* sp., CPC 12171, *Prunus* sp., Canada, EU035420, EU035420, -, -, -, -, -, -; *Knufia* sp., CPC 12172, *Prunus* sp., Canada, EU035421, EU035421, -, -, -, -, -, -; *Knufia* sp., CPC 12173, *Prunus* sp., Canada, EU035422, EU035422, -, -, -, -, -, -; *Knufia tsunedae*, CPC 22931T, Soil, Spain, HG003669, HG003672, -, -, -, -, -, -; *Knufia vaticanii*, CBS 139722T = CCFEE 5939, Travertine, St Peter’s colonnade, Vatican City State, KP791780, KR781068, -, -, -, -, -, -; ***Lithohypha catenulata***, CGMCC 3.14885T, ornamental Limestone, Beijing, China, **JN650519**, **KP174917**, **JN695021**, **KP174961**, **KP226517**, **KP226544**, -, -; ***Lithohypha catenulata***, CGMCC 3.17346, limestone, Zhangjiakou, China, **KP174840**, **KP174951**, **KP174900**, **KP174963**, **KP226518**, **KP226542**, -, -; ***Lithohypha catenulata***, CGMCC 3.14008, ornamental limestone, Beijing, China, **KP174841**, **KP174918**, **KP174902**, **KP174962**, **KP226519**, **KP226543**, -, -; *Lithophila guttulata*, CCFEE 5894, Marble, St Peter’s colonnade, Vatican City State, KP791769, KR781057, -, -, -, -, -, -; *Lithophila guttulata*, CBS 139723T = CCFEE 5907, Marble, Vatican City State, KP791773, KR781061, -, -, -, -, -, -; *Lithophila guttulata*, CCFEE 5908, Marble, (catalogue n. 37107), CP, Vatican City State, KP791770, KR781058, -, -, -, -, -, -; *Metulocladosporiella musicola*, CBS 110960T, *Musa acuminata*, South Africa, DQ008127, DQ008153, -, -, -, -, -, -; *Metulocladosporiella musicola*, CBS113864, *Musa* sp., Uganda, DQ008133, DQ008157, -, -, -, -, -, -; *Metulocladosporiella musicola*, CBS113865, *Musa sapientum*, Uganda, DQ008134, DQ008158, -, -, -, -, -, -; *Strelitziana africana*, ICMP 21760, -, Hawaiian, MK210540, MK210501, -, -, -, -, -, -; *Strelitziana africana*, ICMP 21758, -, Hawaiian, MK210538, MK210501, -, -, -, -, -, -; *Strelitziana albiziae*, CBS 126497T, -, Korea, MH864122, MH875582, -, -, -, -, -, -; *Strelitziana australiensis*, CBS 124778T, *Eucalyptus* sp., Queensland, Australia, GQ303295, GQ303326, -, -, -, -, -, -; *Strelitziana malaysiana*, CPC 24874T, leaf spots on *Acacia mangium*, Malaysia, Sabah, KR476731, KR476766, -, -, -, -, -, -; ***Trichomerium cicatricatum***, CGMCC 3.17344, Rock, Jiangxi, China, **KP174853**, **KP174947**, **KP174897**, **KP174991**, **KP226530**, **KP226541**, -, -; ***Trichomerium cicatricatum***, CGMCC 3.17307T, Rock, Jiangxi, China, **KP174854**, **KP174948**, **KP174898**, **KP174992**, **KP226531**, **KP226540**, -, -; ***Trichomerium deniqulatum***, MFLUCC 10-0884T, Living leaf of *Psidium Guajava*, Thailand, **JX3136554**, **JX313660**, -, -, -, -, -, -; *Trichomerium dioscoreae*, CBS 138870, Leaf of Dioscorea sp., Japan, KP004468, -, -, -, -, -, -, -; ***Trichomerium flexuosum***, CGMCC 3.17988T, Rock, Guizhou, China, **KX348464**, **KX348475**, **KX348484**, **KX348478**, **KX348490**, **KX348496**, -, -; ***Trichomerium flexuosum***, CGMCC 3.17986, Rock, Guizhou, China, **KX348465**, **KX348473**, **KX348482**, **KX348476**, **KX348488**, **KX348494**, -, -; ***Trichomerium flexuosum***, CGMCC 3.17987, Rock, Guizhou, China, **KX348466**, **KX348474**, **KX348483**, **KX348477**, **KX348489**, **KX348495**, -, -; *Trichomerium foliicola*, MFLUCC 10-0078T, Living leaf of *Murray apaniculata*, Thailand, JX313655, JX313661, -, -, -, -, -, -; *Trichomerium foliicola*, MFLUCC 10-0054, *Mangifera indica*, Thailand, JX313651, JX313657, -, -, -, -, -, -; ***Trichomerium lapideum***, CGMCC 3.17311T, Granite, Jiangxi, China, **KP174850**, **KP174946**, **KP174895**, **KP174988**, **KP226528**, **KP226539**, -, -; ***Trichomerium lapideum***, CGMCC 3.17309, Granite, Jiangxi, China, **KP174852**, **KP174944**, **KP174896**, **KP174989**, **KP226534**, **KP226537**, -, -; ***Trichomerium lapideum***, CGMCC 3.17310, Granite, Jiangxi, China, **KP174851**, **KP174945**, **KP174899**, **KP174990**, **KP226529**, **KP226538**, -, -; ***Trichomerium leigongense***, CGMCC 3.17983T, Rock, Guizhou, China, **KX348467**, **KX348470**, **KX348485**, **KX348479**, **KX348491**, **KX348497**, -, -; ***Trichomerium leigongense***, CGMCC 3.17984, Rock, Guizhou, China, **KX348468**, **KX348471**, **KX348487**, **KX348481**, **KX348493**, **KX348499**, -, -; ***Trichomerium leigongense***, CGMCC 3.17985, Rock, Guizhou, China, **KX348469**, **KX348472**, **KX348486**, **KX348480**, **KX348492**, **KX348498**, -, -; *Placocarpus schaereri*, AFTOL 2289, -, -, -, EF643766, -, -, -, -, -, -.
